# Extreme Hypoxic Conditions Induce Selective Molecular Responses and Metabolic Reset in Detached Apple Fruit

**DOI:** 10.3389/fpls.2016.00146

**Published:** 2016-02-16

**Authors:** Dubravka Cukrov, Monica Zermiani, Stefano Brizzolara, Alessandro Cestaro, Francesco Licausi, Claudio Luchinat, Claudio Santucci, Leonardo Tenori, Hans Van Veen, Andrea Zuccolo, Benedetto Ruperti, Pietro Tonutti

**Affiliations:** ^1^Istituto di Scienze della Vita, Scuola Superiore Sant'AnnaPisa, Italy; ^2^Dipartimento di Agronomia Animali Alimenti Risorse Naturali e Ambiente, University of PadovaPadova, Italy; ^3^Centro Ricerca e Innovazione, Fondazione Edmund Mach di San Michele all'AdigeTrento, Italy; ^4^CERM, University of FirenzeFirenze, Italy; ^5^Fondazione FiorGen OnlusFirenze, Italy

**Keywords:** cortex, ethylene-responsive factors, low oxygen stress, *Malus domestica*, metabolic profiling, postharvest, pyruvate metabolism, storage

## Abstract

The ripening physiology of detached fruit is altered by low oxygen conditions with profound effects on quality parameters. To study hypoxia-related processes and regulatory mechanisms, apple (*Malus domestica*, cv Granny Smith) fruit, harvested at commercial ripening, were kept at 1°C under normoxic (control) and hypoxic (0.4 and 0.8 kPa oxygen) conditions for up to 60 days. NMR analyses of cortex tissue identified eight metabolites showing significantly different accumulations between samples, with ethanol and alanine displaying the most pronounced difference between hypoxic and normoxic treatments. A rapid up-regulation of *alcohol dehydrogenase* and pyruvate-related metabolism (*lactate dehydrogenase, pyruvate decarboxylase, alanine aminotransferase*) gene expression was detected under both hypoxic conditions with a more pronounced effect induced by the lowest (0.4 kPa) oxygen concentration. Both hypoxic conditions negatively affected *ACC synthase* and *ACC oxidase* transcript accumulation. Analysis of RNA-seq data of samples collected after 24 days of hypoxic treatment identified more than 1000 genes differentially expressed when comparing 0.4 vs. 0.8 kPa oxygen concentration samples. Genes involved in cell-wall, minor and major CHO, amino acid and secondary metabolisms, fermentation and glycolysis as well as genes involved in transport, defense responses, and oxidation-reduction appeared to be selectively affected by treatments. The lowest oxygen concentration induced a higher expression of transcription factors belonging to AUX/IAA, WRKY, HB, Zinc-finger families, while MADS box family genes were more expressed when apples were kept under 0.8 kPa oxygen. Out of the eight group VII ERF members present in apple genome, two genes showed a rapid up-regulation under hypoxia, and western blot analysis showed that apple MdRAP2.12 proteins were differentially accumulated in normoxic and hypoxic samples, with the highest level reached under 0.4 kPa oxygen. These data suggest that ripe apple tissues finely and specifically modulate sensing and regulatory mechanisms in response to different hypoxic stress conditions.

## Introduction

Hypoxic (reduced oxygen level) or anoxic (complete absence of oxygen) conditions can occur in natural environments when the root system is flooded (waterlogging) or the whole plant is overwhelmed by water (submergence). A wealth of information is available on metabolic (in particular energy metabolism) responses, oxygen signaling mechanisms, and the transcriptional regulation occurring under low oxygen stress concerning the vegetative tissues of model species such as *Arabidopsis* and rice (van Dongen and Licausi, 2014).

A common response among plant species to low oxygen availability is the activation of the fermentative pathways to sustain glycolysis in the absence of mitochondrial respiration, which results in the production of lactate or ethanol. The accumulation of alanine, γ-aminobutirate (GABA), and succinate and a decrease in the amount of aspartate appear to be other common responses (Narsai et al., 2011). Transcription factors (TFs) such as zinc-finger, ethylene responsive factors (ERFs), and LOB domain proteins have also been identified as highly induced by hypoxia in various species (Mustroph et al., 2010). In *Arabidopsis*, transcriptional adjustments to oxygen depletion are triggered by the ERF family group VII, which may act as an oxygen sensor in plants through the N-end rule pathway (NERP) for protein degradation (Gibbs et al., 2011; Licausi et al., 2011a; Licausi, 2012).

Apart from the common responses, which have been identified as conserved throughout the plant kingdom but also in different plant tissues and cell types (Mustroph et al., 2009, 2010, 2014), a large array of tissue and cell type specific hypoxic responses have been identified (Mustroph et al., 2009). Intrinsic differences in metabolism between organs have also been detected (Ellis et al., 1999), and the severity of oxygen shortage can induce varying responses. An analysis of large sets of global expression data highlighted significant differences in the oxidative stress components in *Arabidopsis* under anoxia and hypoxia (Pucciariello et al., 2012).

A reduction in oxygen levels (together with an increase in carbon dioxide and a decrease in temperature) is deliberately applied to extend the storage life of fruit in systems called controlled atmosphere (CA; Yahia, 2009). This technology leads to a reduction in respiration, as well as ethylene biosynthesis and action, and subsequently to a better maintenance of commercial quality parameters. Pome fruit (apples and European pears) can be successfully stored for several months, for up to 1 year for specific apple varieties under CA conditions where oxygen is generally maintained at concentrations ranging from 2-3 to 1 kPa. In recently introduced advanced CA protocols, oxygen levels are set at concentrations as low as 0.4 kPa (Dynamic CA, DCA). Under DCA apples better maintain technological quality parameters, namely flesh firmness and acidity, the most important drivers of consumer preference for apples, with a reduction in the incidence of cold storage disorders such as superficial scald (Tonutti, 2015). However, in DCA protocols the reduction in oxygen reaches the lowest level tolerated by the fruit, the so-called anaerobic compensation point, with a high risk of severe quality losses due to the onset of internal physiological disorders and the development of off-flavors. Thus, an evaluation of the metabolic conditions of the fruit is crucial in order to quickly adjust the oxygen concentration up to a “safe” level of around 1 kPa, which is above the anaerobic compensation point.

Despite the commercial applications of CA, the precise mode of action of low oxygen in fruit is scarcely understood. Works based on metabolomics (Pedreschi et al., 2009a; Vandendriessche et al., 2013; Hatoum et al., 2014), proteomics (Pedreschi et al., 2007, 2009b), and transcriptomics (Mellidou et al., 2014) have provided important information on the processes and metabolites associated with the development of common physiological disorders in apples and pears (internal browning, core breakdown) during traditional CA storage. These studies reported data on the effects of long-term storage (in general after 4–8 months) at oxygen concentrations ranging from 1 to 3 kPa. Knowledge of the molecular and metabolic responses occurring under extremely low (<1 kPa) oxygen concentrations such as those used in DCA protocols, in particular at the initial stages of the storage period, is very limited. To our knowledge, no information currently exists on the regulatory mechanisms involved in the responses of fruit to hypoxic conditions.

In this work we report data on the application of an integrated approach on apples kept under two levels of low oxygen concentrations throughout the initial 2 months of storage, when, in general, a strict control of the effects of the extremely low oxygen concentration is required for a successful DCA performance.

## Materials and methods

### Plant material and treatments

Apple (*Malus domestica* Borkh., cv Granny Smith) fruit were harvested at commercial ripening stage (starch index of 7, based on a 1–10 scale) in an orchard located at Andriano (Bolzano, Italy). Homogeneous fruits in terms of size and peel color were selected and immediately stored in three refrigerated (1°C) experimental chambers under normoxia. After 3 days of acclimation of fruit to low temperature (T0 sampling point), 0.4 (0.4ox), and 0.8 (0.8ox) kPa oxygen atmospheres (in nitrogen and 0.9–1 kPa CO_2_) were applied in two different chambers, whereas control apple samples were kept under normoxic conditions (Nox). The temperature of the chambers was maintained at 1°C throughout the experimental period (60 days). On day 30, based on ethanol accumulation, (which reached the threshold value of about 75 mg/L of fruit juice in 0.4ox samples), the oxygen concentration in the 0.4 kPa chamber was increased to 0.8 kPa and maintained up to the end of the experimental period. Fruit samples were collected after 3, 10, 17, 24, 31 (1 day after the shift to 0.8 kPa in 0.4ox treatment), and 60 (end of experiment) DIA (days in atmospheres) from T0. The small experimental chambers were equipped with a device allowing fruit sampling without affecting the atmosphere composition. Figure S1 shows the trial and sampling collection times.

Slices (about 1 cm thick) of cortex tissue, isolated from the equatorial part of each of the three fruit (*n* = 3) collected at each sampling date, were immediately frozen in liquid nitrogen and stored at −80°C. Considering differences between the inner and the outer parts of the cortex that have been observed in terms of composition (pears, Pedreschi et al., 2009a) and oxygen concentration (apple, Mellidou et al., 2014), the samples collected included both parts.

### NMR analysis

NMR analysis was performed on a total of 57 samples (T0+18 samples for each treatment). Three biological replicates, each with two technical replicates were analyzed for each sampling time. For each biological replicate 5 g of frozen pulp were thawed and homogenized. The juices obtained were immediately filtered through a 0.2 μm PTFE membrane filter and then centrifuged at 14,000 rpm, for 10 min at 4°C. Five hundred and forty microliters of surnatant were mixed with 60 μl of sodium phosphate buffer (0.2 M NaH_2_PO_4_ in 100% ^2^H_2_O, 10 mM TSP, and 30 mM NaN_3_, pH 7.0). A total of 450 μl of the mixture was pipetted into 4.25 mm NMR tubes (Bruker BioSpin srl).

For the analysis of metabolite content, all resonances of interest were manually checked. Signals were assigned on template one-dimensional NMR profiles using matching routines of AMIX 7.3.2 (Bruker BioSpin) in combination with the BBIOREFCODE (Version 2-0-0; Bruker BioSpin) reference database and specific published literature (Vermathen et al., 2011). Each identified signal (i.e., the corresponding peak in the NMR spectrum) was automatically integrated (i.e., the area under the curve was calculated, being directly proportional to the total concentration of the respective metabolite) using a homemade R-script. No calibration using reference standards was performed, thus, the exact concentration of each metabolite was not determined but only the relative intensity is reported.

### RNA extraction and transcript analyses

Total RNA was extracted as described by Botton et al. (2011). RNA quality and integrity were checked on a Bioanalyzer 2100 (Agilent, Santa Clara, CA). Reverse transcription and RT-qPCR analyses were conducted as described by Falchi et al. (2010). Gene selective primers were constructed on putative 3′ untranslated regions (UTR), using multiple sequence alignments to select non conserved regions (CLUSTALX, http://www.ebi.ac.uk/Tools/clustalw/index.html). Primers used for RT-qPCR were designed with Primer3; (Rozen and Skaletsky, 2000; http://frodo.wi.mit.edu/primer3/), tested with Oligonucleotide Properties Calculator (Kibbe, 2007; http://www.basic.northwestern.edu/biotools/OligoCalc.html) and the PRaTo tool (Nonis et al., 2011; http://prato.daapv.unipd.it) to identify the best selective pairs. Specificity was confirmed by melting curve analysis. Data were processed with DataAssist Software version 2.0 (Applied Biosystems, Monza, Italy) and normalized to Md_8283:1:a (Botton et al., 2011) using the (Livak and Schmittgen, 2001) method. RT-qPCR analyses were performed on three biological replicates for each sampling time. List of genes analyzed and primers are reported in Table S1.

Transcripts of three samples with three biological replicates were analyzed by RNAseq: T0 (A), 0.4ox at 24 DIA (B), 0.8ox at 24 DIA (C). The 24 DIA samples were chosen as they represent the latest samplings under the two different hypoxic conditions before the shift to 0.8 kPa oxygen of the 0.4ox samples. Sequencing libraries were prepared using the TruSeq RNA Sample Prep kit (Illumina Inc.) according to the manufacturer's instructions. Sequencing was carried out on an Illumina HiSeq2000 in two lane single-read 50 bp. Downstream steps including the processing of fluorescent images into sequences, base-calling, and overall quality value calculations were performed using the Illumina data processing pipeline version 1.8.2.

### RNA-seq data processing and analysis

Contaminants and low quality regions were removed from illumina reads using the tool ERNE (Del Fabbro et al., 2013). Tophat2 (Kim et al., 2013) was used to align the reads onto the reference genome (Mdomestica_196_v1.0; www.phytozome.org) previously indexed with Bowtie2 (Langmead and Salzberg, 2012). To avoid ambiguous read mapping, TopHat2 was used enabling the option -M that removes reads with multiple mapping locations. The reference annotation for gene models was downloaded from www.phytozome.org. No attempt for *de novo* transcript reconstruction was carried out. Cuffdiff (Trapnell et al., 2012) was used to: determine the amount of reads mapping in different locations, normalize the counts as Reads Per Kilobase of exon per Million fragments mapped -RPKM- (Mortazavi et al., 2008), compare the expression between two different conditions, and assess the significance of changes (expressed as Log_2_ fold ratios). For data visualization, MapMan (Usadel et al., 2009) was used to place differential gene expression data in the context of the appropriate metabolic pathways. The appropriate mapping filewas retrieved from http://mapman.gabipd.org/. A GO analysis was performed with the Bioconductor GOseq package correcting for gene length bias (Young et al., 2010), using DE genes with |logFC|>1 and |qval|<0.001. GO annotation was obtained using Blast2Go (Conesa and Götz, 2008). The correspondence between MDP release numbers cited in the manuscript and gi NCBI is reported in Table S2.

### Protein extraction and western blotting

For each biological replicate, 1.5 g of frozen tissue was ground in liquid nitrogen to a fine powder with a mortar and pestle. Total protein was extracted with a hot SDS buffer followed by ice-cold TCA/acetone precipitation, as described by Song et al. (2006). For each sample, a total of 10 μg of proteins was resolved in 8% acrylamide SDS-PAGE gel followed by western blotting according to standard protocols. Rabbit polyclonal anti-MdERF antibodies were used, that had been raised against three MdRAP2.12 specific synthetic peptides (LTADYLWPDLKKPSS, VNDSSQDYYSALGFL, and GEQGSKTPEISSVL, displayed by Mdo006712 and Mdo006699; Licausi et al., 2011a) and affinity purified at Primm Srl laboratories (Milan, Italy). Anti-rabbit secondary antibody was provided by Sigma (Milan, Italy). Western blotting experiments were repeated with three different biological samples.

### Statistical analyses

**Figure 1:**
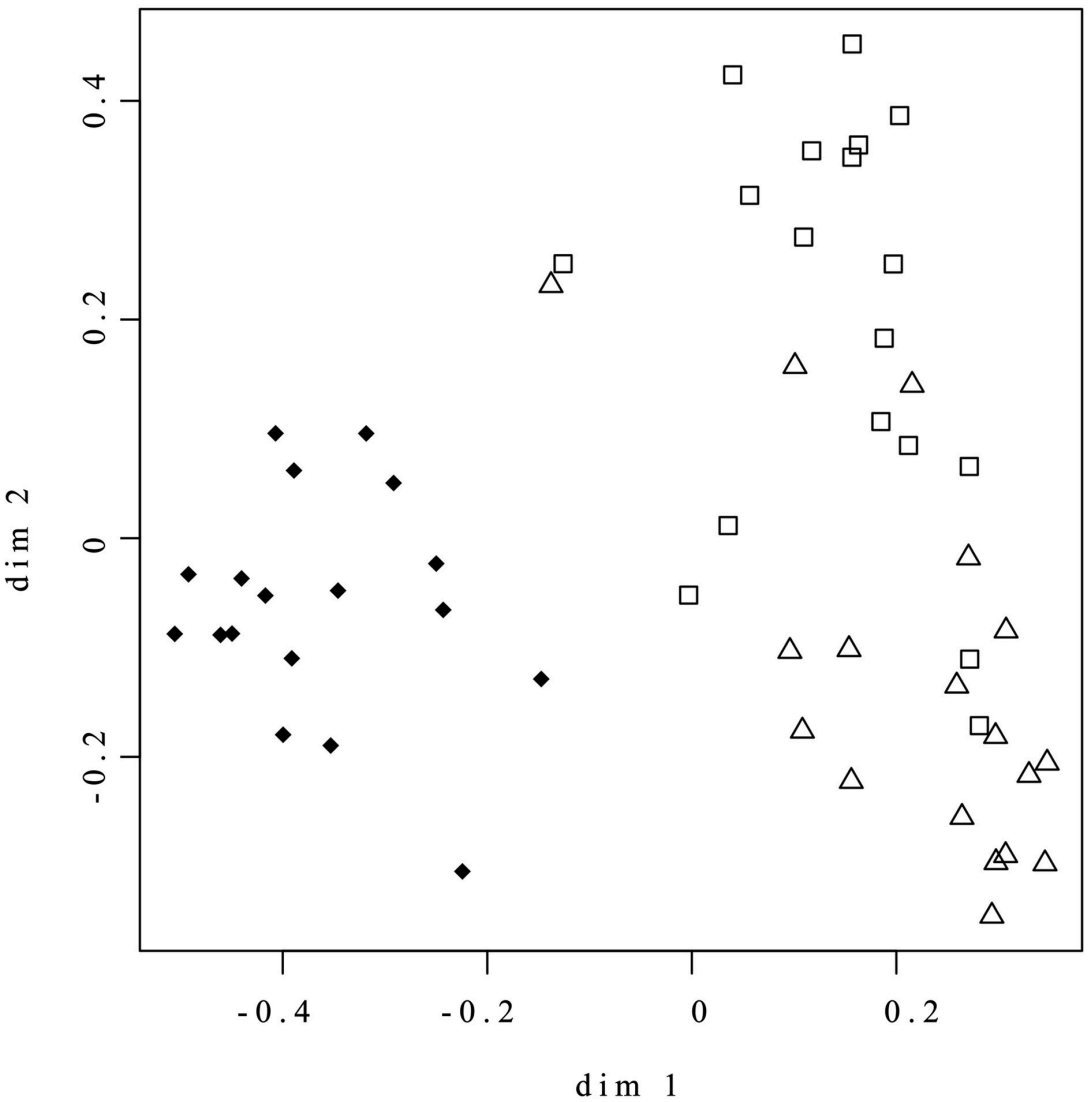
**Random Forest Unsupervised analysis of whole set of NMR data**. ♦ = Nox; Δ = 0.4ox; □ = 0.8ox. The axes represent the coordinates of the points in the Euclidean space reconstructed from the Random Forest proximity matrix, as detailed in the Materials and Methods Section. Similar samples, i.e., samples with similar metabolic content, tend to be tightly clustered.

Random Forest was applied for both supervised (outcome labels are used) and unsupervised (outcome labels are not used, i.e., the systems is not trained to recognize different conditions) learning, using sum-normalized bins as input variables (Breiman, 2001). Briefly, the algorithm fits many classification trees to a data set, and then combines the outcomes from all the trees. The algorithm begins with the selection of many (2000 in our setting) bootstrap samples from the original data. A tree is fitted to each bootstrap sample. Because many different trees are built on the same dataset, the RF algorithm allows to derive a similarity measure between all pairs of samples by counting the number of times two samples are placed in the same terminal node of a tree. The matrix that contains all the pairwise proximities between samples (proximity matrix) can be considered as a matrix of distances between points in an Euclidean space. By means of classical multidimensional scaling, the coordinate of the point can be calculated from proximities and the points (representing the samples) can be plotted to have an overview of the space distribution of the data. In the unsupervised case, this plot is extremely useful to enlighten the presence of natural partitions or cluster among the data. The scaling coordinate for the unsupervised analysis are shown in Figure 1. The estimate error rate for the supervised analysis (discrimination between different hypoxia conditions) is 11%.

The significance (*p* < 0.05) of increasing/decreasing tendencies of metabolite levels in the three storage conditions was assessed by the Wilcoxon signed-rank test (Wilcoxon, 1945).

The association between oxygen concentration and the gene expression level was addressed by analysis of variance (ANOVA). For ANOVA, mean delta *C*_*T*_ values were calculated for each oxygen condition and pairwise comparisons of mean expression levels were made between different oxygen conditions. The pairwise comparisons were summarized using the least-significant difference approach. The fold change scores (calculated using the 2^−ΔΔCt^ method) for all examined cases were presented as a graph using error bars with 95% confidence intervals (CIs). Statistical significance was established by using a conventional *P* level of 0.05.

The concordance of RNA-seq and the RT-qPCR data was tested using the Pearson correlation (Figure S2). ANOVA was performed to test differences in regression lines.

The RNAseq data set has been deposited into NCBI under accession GSE72153.

## Results

### Hypoxia-related metabolic processes in apple cortex

NMR analysis was used to study the overall effect on the metabolic profiles of apple fruit induced by maintenance under low oxygen conditions for up to 60 days. The Random Forest Unsupervised analysis of the whole set of NMR Spectral data clearly discriminated between the control (Nox, samples kept in normoxia) and those maintained under hypoxic conditions (0.4 and 0.8 kPa oxygen atmospheres, 0.4ox and 0.8ox samples, respectively; Figure 1). In the score plot, Nox samples were well separated from the hypoxic samples along Dimension 1 highlighting the marked differences induced throughout 60 days of hypoxia in the metabolic profile of the apple cortex. Alanine (doublet, detected at 1.48 ppm), asparagine (doublet, 2.94 ppm), aspartate (multiplet, 2.87 ppm), and ethanol (triplet, 1.18 ppm) contributed the most to the separation between normoxic and hypoxic samples. On the other hand, 0.8ox samples were only partially separated from the other two groups along Dimension 2 (Figure 1).

After manually checking the resonances of interest, 16 metabolites were identified, of which eight were differentially accumulated between one or both hypoxic samples and the control in terms of the whole set of samples from T0 to 60 Days In Atmosphere (DIA; Table 1). Ethanol and alanine significantly increased under both hypoxic conditions, with ethanol displaying a higher accumulation in 0.4ox than in 0.8ox (log_2_ FC of 3.93 and 0.85 compared to Nox, respectively). Uridine showed a significantly lower accumulation in both hypoxic conditions (log_2_ FC of −1.03 and −0.83 in 0.4ox and 0.8ox, respectively), whereas other compounds displayed different trends, such as threonine and GABA, which were significantly higher only in 0.4ox and 0.8ox samples, respectively. Asparagine and aspartate showed a −0.44 fold change in 0.4ox samples, whereas for α-galactose a slight decrease was detected only in 0.8ox samples. No significant changes were detected for valine, sucrose, fructose, α-glucose, β-galactose, isoleucine, leucine, and fucose (Table 1).

**Table 1:** **Identified compounds by NMR analysis**.

**Compounds**	**0.4ox vs. Nox**	**0.8ox vs. Nox**
Ethanol	3.93^*^	0.85^*^
Aspartate	−0.44^*^	−0.1
Alanine	0.78^*^	0.73^*^
Asparagine	−0.44^*^	−0.11
GABA	0.26	0.12^*^
Isoleucine + Leucine	0.16	0.02
Threonine	0.19^*^	0.02
Valine	0.18	0.09
Uridine	−1.03^*^	−0.83^*^
Fructose	0.02	0
Fucose	0.12	0.06
α-galactose	−0.05	−0.08^*^
β-galactose	−0.9	−0.07
α-glucose	−0.07	0.01
Sucrose	−0.04	−0.06

Fold changes of selected compound accumulation in 0.4ox and 0.8ox samples compared to control (Nox). NMR data of all sampling points (from T0 to 60 DIA) have been considered. Values in bold^*^ are significant at p < 0.05. The fold change was calculated according to the following formula: FC = −log_2_ (median 0.4ox or 0.8ox/median Nox).

**Figure 2:**
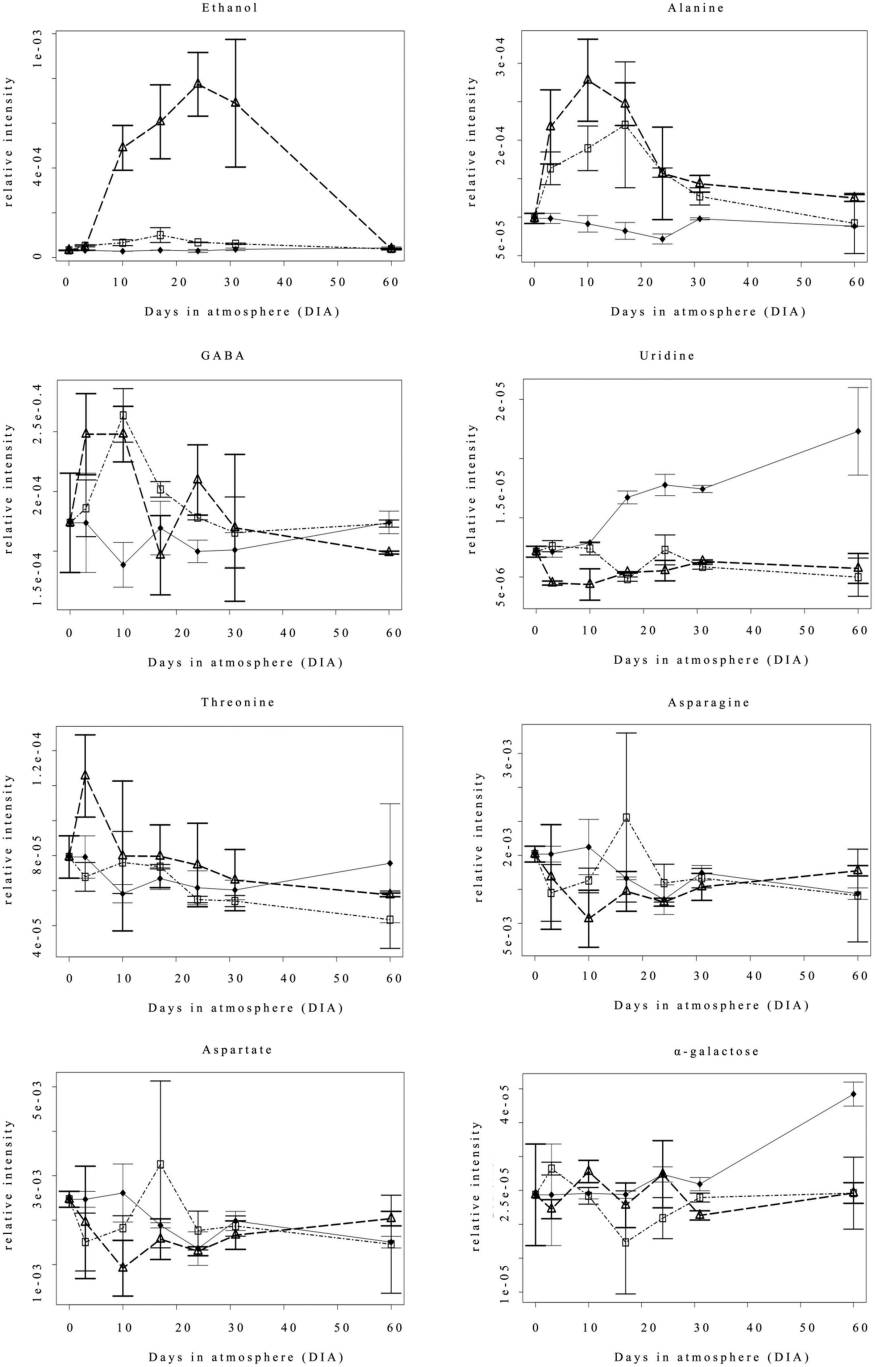
**Changes in the accumulation (expressed as relative intensity) of 8 metabolites detected by NMR analysis throughout the experimental period**. Each point represents the median value of three analyses; bars indicate the Median Absolute Deviation (MAD). ♦ = Nox; Δ = 0.4ox; □ = 0.8ox. For the 0.4ox sample 31 DIA corresponds to 1 day after the shift from 0.4 to 0.8 kPa oxygen.

Figure 2 shows the accumulation (relative intensity) time course of the eight significantly different compounds reported in Table 1. Ethanol remained at basal levels in the Nox samples throughout the experimental period, and differences were observed starting from 10 DIA, when 0.4ox samples reported a marked accumulation of the compound. Under such hypoxic conditions, the level of ethanol remained high, reaching the highest values at 24 DIA, followed by a slight decrease at 31 DIA (1 day after the shift from 0.4 to 0.8 oxygen concentration). 0.8ox samples were characterized by a lower but statistically significant increase in ethanol concentration at 10, 17, and 24 DIA compared to the Nox control. No differences among samples were detected at the end of the experimental period (60 DIA). Alanine (Figure 2) content showed a similar behavior in both hypoxic conditions, with a rapid increase at 3 DIA, reaching the highest resonance values at 10 DIA in 0.4ox sample followed by a decreasing trend. A significant increase in GABA levels was observed in 0.4ox samples immediately after 3 DIA, when 0.8ox samples still showed no difference from the control (Figure 2). At 10 DIA samples maintained under both hypoxic conditions were characterized by high GABA levels, which afterwards started to decline. An interesting trend was observed concerning the evolution of uridine which showed increasing levels starting at 17 DIA in the Nox apples. Conversely, in both hypoxic samples uridine did not show a marked change throughout the experimental period, with the exception of a slight transient decrease in 0.4ox up to 10 DIA (Figure 2).

Threonine and asparagine (Figure 2) remained rather stable and did not differ amongst samples, except for a significant increase at 3 DIA and a significant decrease at 10 DIA, respectively, in 0.4ox samples. The aspartate trend was similar to that of asparagine. A similar behavior of α-galactose was observed in the three different samples throughout the experimental period, with the exception of the last sampling (60 DIA) when a significant increase was observed in apples kept under normoxia.

Based on these metabolomics analyses and the information available in model species concerning hypoxia-affected genes and processes, nine genes were selected for a detailed analysis of expression (RT-qPCR) throughout the experimental period, to characterize apple cortex responses and the progression of ripening in the presence of different oxygen regimes.

**Figure 3:**
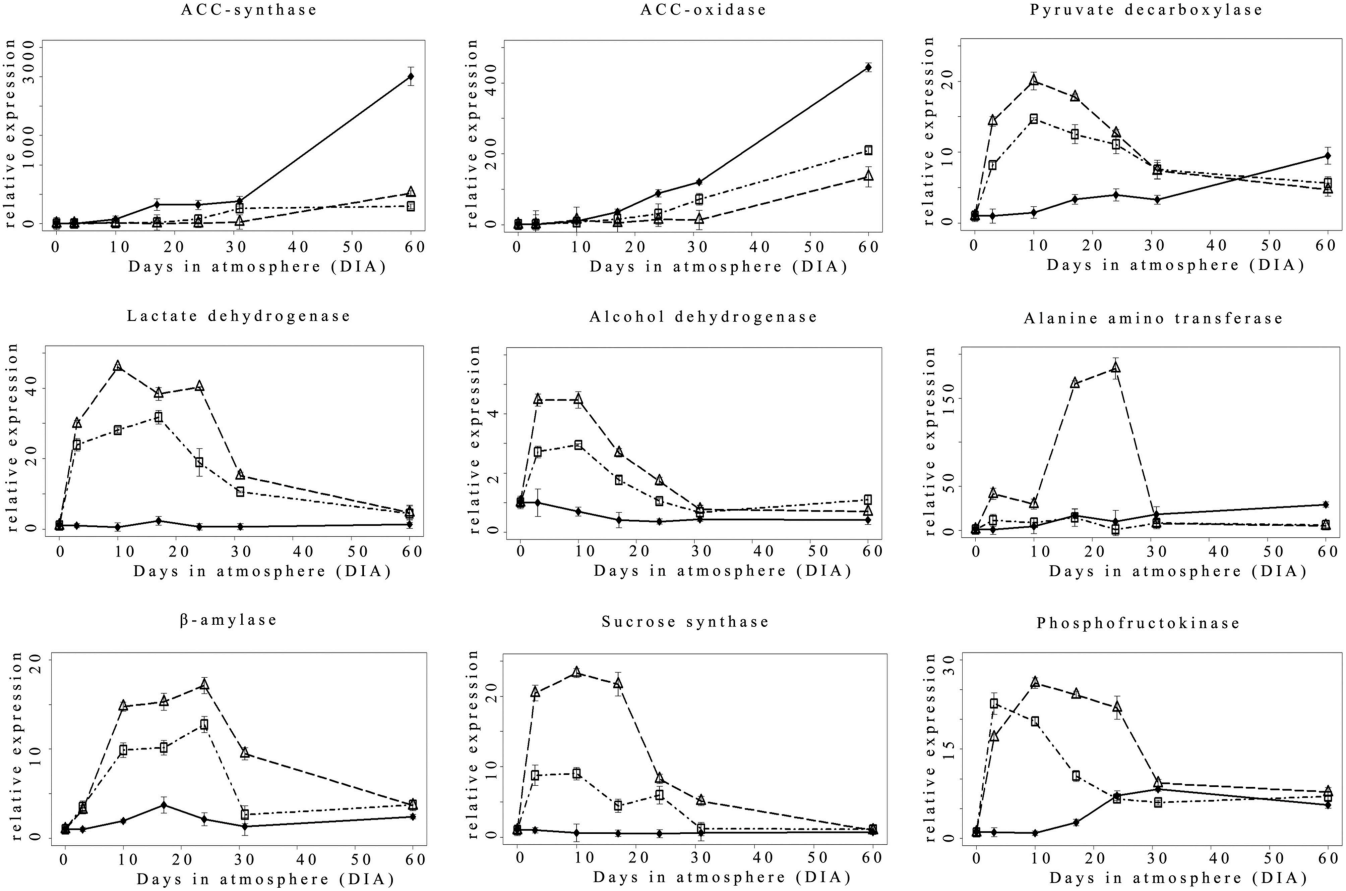
**Relative (T0 value = 1) expression pattern of selected hypoxia-responsive genes ♦ = Nox; Δ = 0.4ox; □ = 0.8ox**. Data are means of three biological replicates. Error bars represent 95% confidence intervals. For the 0.4ox sample 31 DIA corresponds to 1 day after the shift from 0.4 to 0.8 kPa oxygen.

Considering ethylene biosynthesis, the expression pattern of the main apple ripening-related *ACC-synthase* (*ACS*) and *ACC oxidase* (*ACO*) genes showed an increasing expression trend in Nox samples starting from 17 DIA with the highest values reached at the end of the experimental period (60 DIA; Figure 3). Hypoxic conditions (0.4ox and 0.8ox) negatively affected transcript accumulation for both genes: in 0.8ox samples, the onset of *ACS* and *ACO* transcriptional up-regulation was delayed up to 24 DIA, and further shifted to 31 DIA in the 0.4ox samples.

The expression pattern of genes involved in fermentative metabolism is known to be highly responsive to oxygen levels. In fact a rapid up-regulation of *pyruvate decarboxylase (PDC), lactate dehydrogenase (LDH), and alcohol dehydrogenase* (*ADH*) was detected at 3 DIA, peaking between 3 and 10 (*ADH*) and 10 and 17 DIA (*PDC, LDH*) in both 0.4ox and 0.8ox, with the former sample showing a higher induction of specific transcript accumulation (Figure 3). After the peak, *ADH, PDC*, and *LDH* transcripts leveled off in both hypoxic conditions progressively reaching values comparable between the two hypoxic samples and more similar to those found in the control, at 31 and at 60 DIA, respectively. Interestingly, in the Nox sample, *PDC* expression showed an increasing trend after 17 DIA throughout the experimental period, similarly to *ACS* and *ACO* genes, thus possibly in relation to the slow evolution of ripening under normoxic conditions.

A different pattern of expression was observed for an *alanine aminotransferase* (*AlaAT*) gene. In fact, there was a dramatic increase at 3 and 10 DIA in 0.4ox samples, which showed the highest expression levels at 17 and 24 DIA. A marked decrease in transcript accumulation was then observed at 31 DIA, 1 day after oxygen levels were shifted from 0.4 to 0.8 kPa, when differences between hypoxic samples became undetectable. In 0.8ox samples, *AlaAT* gene expression appeared to remain at basal levels throughout the experiment, while there was a slight increase for Nox samples starting from 17 DIA onwards.

An expression behavior similar to that described for *PDC, LDH*, and *ADH* genes, was also observed for three genes involved in sucrose/starch metabolism encoding β-amylase (β -amy), sucrose synthase (*SuSy*), and phosphofructokinase (*PFK*; Figure 3). An up-regulation of expression was detected for all three genes starting from 3 DIA in both hypoxic samples compared to the control, with a peak of expression observed at 10 DIA (*SuSy* and *PFK*) and at 24 DIA (β-amy). 0.4ox samples always showed the highest transcript levels, while in 0.8ox samples there was a similar expression trend with lower relative levels. Also for *SuSy, PFK*, and β-amy genes, a decrease in transcript accumulation was observed in both hypoxic samples after the peak of maximal expression, progressively leading to similar levels in all oxygen regimes at the end of the experimental period (60 DIA). No significant changes of expression were detected in the Nox sample, where basal expression levels were maintained with the exception of a slight increase occurring at 17 DIA for β-amy, and an increasing trend, starting after 17 DIA onwards, taking place for the *PFK* gene (Figure 3).

### Apple ERFs and oxygen-sensing mechanisms

**Figure 4:**
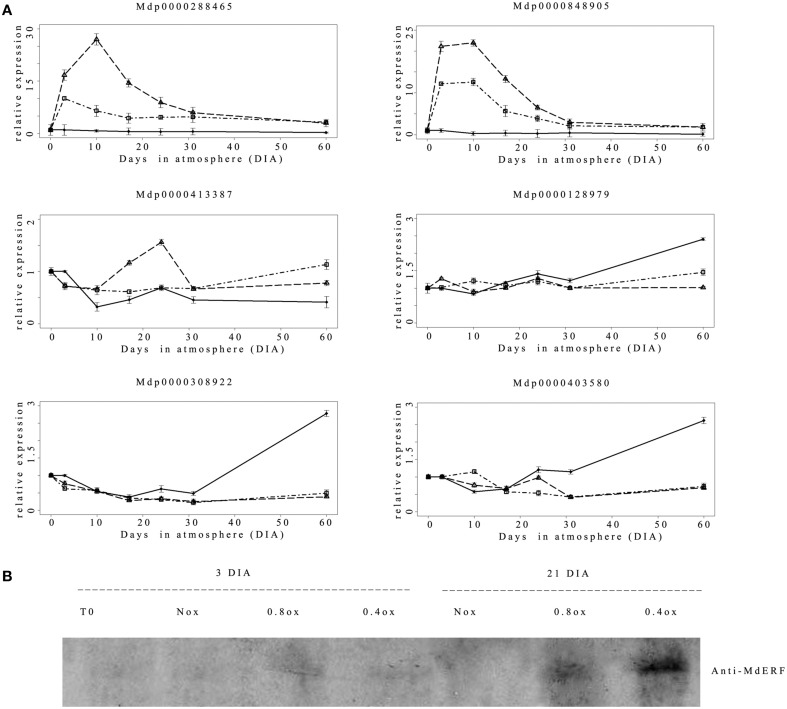
**(A)** Relative (T0 value = 1) expression pattern of the 6 apple class VII ERF genes. ♦ = Nox; Δ = 0.4ox; □ = 0.8ox. Data are means of three biological replicates. Error bars represent 95% confidence intervals. For 0.4ox samples 31 DIA corresponds to 1 day after the shift from 0.4 to 0.8 kPa oxygen. **(B)** Western blot analysis of apple class VII ERFs protein abundance carried out with specific antibodies raised against MDP308922 and MDP403580. The western blot has been performed on three biological replicates with similar results.

In *Arabidopsis*, hypoxia-responsive gene expression is regulated through the fine-tuned mechanism involving specific TFs belonging to group VII ERFs (Bui et al., 2015). These are degraded under normoxic conditions and stabilized under hypoxia via the so-called N-end rule (NERP) pathway. This stabilization triggers hypoxic specific gene expression (Gibbs et al., 2011; Licausi et al., 2011a). The apple genome contains eight genes coding for ERF VII TFs (Girardi et al., 2013), distinguished by a conserved N-terminal consensus MCGGAI. Based on this information, six different members of the apple group VII ERF were analyzed in terms of expression pattern throughout the experimental period (Figure 4A). Three genes (*MDP0000128979, MDP0000403580, and MDP0000308922*, putative orthologous of *Arabidopsis* RAP2.12) displayed a similar trend remaining essentially at basal levels in both hypoxic samples with a slight or no difference among treatments until the last sampling date (60 DIA). The same genes however, displayed a progressive up-regulation in the Nox sample starting from 24 to 31 DIA. One gene (*MDP0000413387*) showed a slight transient increase in expression at 24 DIA in the 0.4ox sample, whereas two other members (*MDP0000288465* and *MDP0000848905*) displayed a marked and rapid increase in transcript accumulation which started as early as 3 DIA in the two hypoxic conditions. Higher levels of transcript accumulation were detected in the 0.4ox sample, where they reached the highest expression at 10 DIA, followed by a steady decrease. No differences between 0.8ox and 0.4ox were observed starting from 31 DIA.

Western blot analysis carried out with antibodies raised against peptides specific to the putative apple proteins orthologous to the oxygen sensors AtRAP2.12 and AtRAP2.2 (MDP0000308922 and MDP0000403580) revealed the absence of a detectable signal at T0 in the Nox sample, while at 3DIA a faint band could be detected in both hypoxic samples (Figure 4B). A marked accumulation of the specific proteins was observed at 24 DIA under hypoxic conditions, more abundantly in 0.4ox and to a lesser extent in 0.8ox (Figure 4B).

### Transcript profiling of the apple cortex under different hypoxic conditions

**Figure 5:**
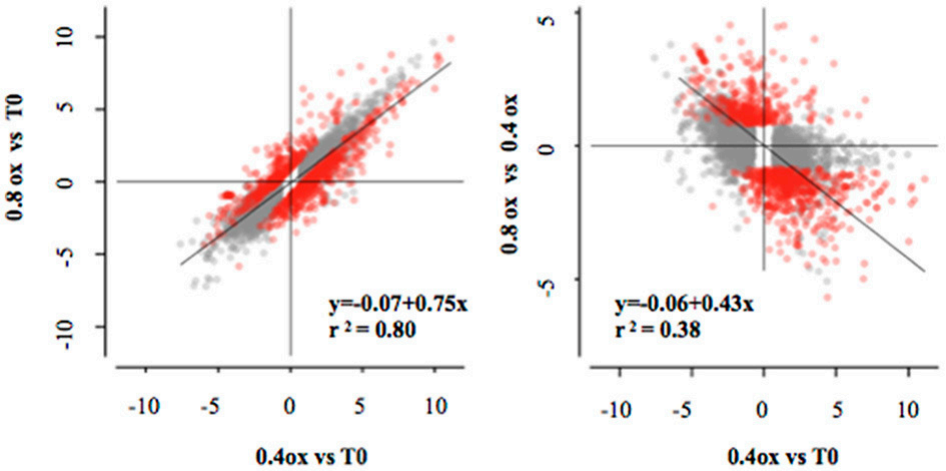
**Scatterplot of the log_**2**_ FCs from all identified differentially expressed genes in all comparisons (Qval < 0.001)**. Red dots are genes differentially expressed between the 0.8ox and 0.4ox samples (Qval < 0.001). Linear regression is based on all DEGs (left panel) and the DEGs between 0.4ox and 0.8ox samples (right panel).

Apple fruit physiology was strongly affected by the oxygen levels during storage. The analysis of hypoxia-related metabolic profiles showed a clear difference in ethanol between O_2_ levels, and targeted gene expression profiling. Western blot analysis confirmed a strong effect of different treatments on ERF abundance. Thus, the physiological and biochemical changes in fruit under low oxygen storage are likely to require significant transcriptional reconfiguration. Consequently, in order to identify novel processes characterizing storage under different O_2_ conditions, the apple cortex was transcriptionally profiled. Illumina RNAseq of the samples A (T0), B (0.4ox at 24DIA), and C (0.8ox at 24 DIA) generated more than 126 million short single-end reads, with each cDNA library containing more than six millions reads, of which more than 88% were mapped (Table S3). In addition, log_2_-FC measured by RNA-Seq and RT-qPCR were, taken together, significantly indistinguishable (*p* > 0.36, ANCOVA; Figure S2). A total of 4989, 4896, and 1034 genes were identified as differentially expressed when comparing different samples (0.4ox vs. T0, BvsA; 0.8ox vs. T0, CvsA; and 0.8ox vs. 0.4ox, CvsB, respectively; *q* < 0.001, Table S4). Overall, the effect of storage was similar for both oxygen concentrations, as demonstrated by the strong correlation between the transcriptional change over time for both storage at 0.8 and 0.4 kPa oxygen (*R*^2^ = 0.80, Figure 5).

The nature of the overall transcriptional change over time during storage was characterized by Gene Ontology (GO) enrichment analysis. Amongst genes that were up-regulated under both 0.8 and 0.4 kPa oxygen conditions compared to T0 samples (Table S5) a strong overrepresentation was found for a range of categories associated with biological processes such as responses to biotic stimulus, oxidation-reduction, and amino acid biosynthetic processes, defense response, glycolytic, and sucrose metabolic processes. Considering molecular functions, intramolecular lyase, cysteamine dioxygenase, oxidoreductase, and protein kinase activity, as well as iron and magnesium ion binding were the overrepresented GO categories detected in hypoxic apples.

Concerning the down-regulated genes in both hypoxic samples compared to T0 (Table S6) oxidation-reduction and cellular metabolic processes, steroid biosynthetic and transmembrane transport, and respiratory gaseous exchange were highly overrepresented biological processes (BP). Protein complex and membrane, oxidoreductase activity, and coenzyme binding had the highest *p*-values considering cellular components (CC) and molecular functions (MF), respectively.

Despite the overlap between 0.4ox and 0.8ox levels, more than 1000 genes were identified as differentially expressed between the two hypoxic conditions (*q* < 0.001). A negative correlation between the effect of 0.8 vs. 0.4 kPa oxygen concentration and the response to 0.4 kPa oxygen storage was observed, suggesting a bias exerted by O_2_ levels on the expression dynamics over time (Figure 5). In fact, taking the transcriptome as a whole, storage at 0.8 kPa O_2_ tended to have a less severe effect on the transcriptomic change over time than the 0.4 kPa O_2_ condition. A total of 897 genes were differentially expressed (qth < 0.001 and |fth|> 1) when comparing B vs. C samples, with 351 more expressed in C (0.8ox after 24 DIA) and 546 more expressed in B (0.4ox after 24 DIA; Table S4). MapMan software was used to classify these differentially expressed genes (DEGs) into a set of hierarchical functional categories.

**Figure 6:**
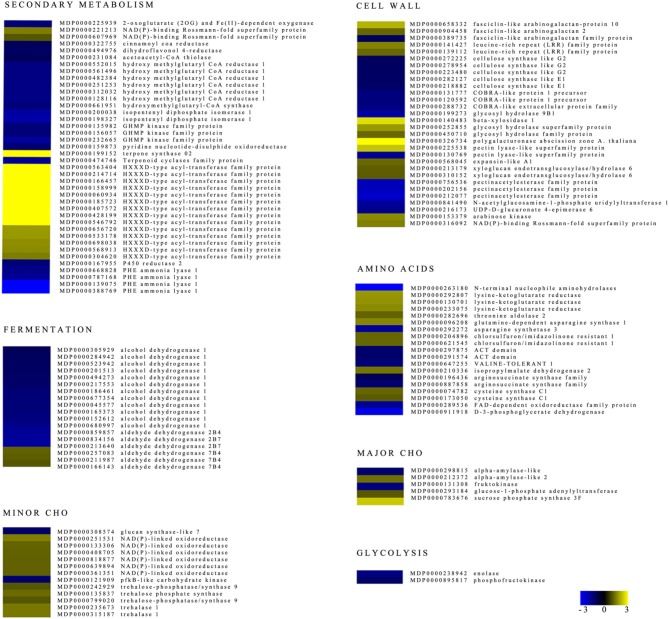
**Heat maps of differentially expressed genes involved in secondary metabolism, cell wall, fermentation, glycolysis, amino acid metabolism, minor and major CHO**. Negative and positive values indicate those genes that showed higher expression in B (0.4ox) or C (0.8ox) samples after 24 DIA, respectively.

The physiology and storability of apples under low oxygen conditions are known to be characterized by changes in the central carbon metabolism with particular emphasis on fermentation, as well as in the cell wall, amino acid, and secondary metabolisms. Identified DEGs assigned to these key-processes were highlighted in the comparison 0.4ox vs. 0.8ox at 24 DIA (Figure 6).

A total of 16 genes involved in pectin esterification (three pectinacetyl esterases) and degradation (pectin lyase), cellulose synthase, and cell wall degradation were more expressed in the B (0.4ox) than the C (0.8ox) sample. Genes encoding a polygalacturonase (showing one of the highest FC values, 4.47), a pectin lyase, a beta xylosidase, two xyloglucan endotransglucosylase, an expansin, and fascilin-like arabinogalacatan and leucine-rich repeat (LRR) proteins were more expressed in the 0.8ox sample.

Specific secondary metabolic processes appeared to be selectively affected by the difference in oxygen concentration after 24 days of storage. Four putative Phe ammonia lyase (*PAL*) genes were markedly more expressed in the 0.4ox samples, as well as a dihydroflavonol 4-reductase, a cinnamoyl CoA reductase, and a number of genes involved in the mevalonate pathway. A marked induction (FC > 3) was observed for terpene synthase as well as for a high number of acyl-transferase genes (nine of them with FC > 3) in the 0.8ox sample.

Considering minor CHO, out of a total of 13 DEGs, 11 (including trehalase and trehalose-phosphatase) were more expressed in the 0.8ox sample, where a sucrose phosphate synthase gene (major CHO) was also highly induced. Samples kept at 0.4 kPa O_2_ showed a higher expression of a putative glucan synthase-like 7, carbohydrate kinase, and fructokinase. Interestingly, two putative alpha-amylase-like genes (*MDP0000131308* and *MDP0000212372*) showed opposite expression trends in 0.4ox and 0.8ox samples.

A total of 19 DEGs were grouped under amino acid bin, indicating that the AA metabolism is highly affected by slight changes in oxygen concentration. Genes involved in asparagine, serine, aspartate and glutamate metabolism, and acetolactate synthase were identified as differentially expressed. Two genes (enolase and phosphofructokinase) involved in glycolysis were highly induced in the 0.4ox sample, where there was also a marked induction in the expression of several *ADH* genes. In the same sample, a higher expression of three aldehyde dehydrogenase (*ALDH*) genes was also observed. Interestingly, three *ALDH* genes (*MDP0000166143, MDP0000211987, MDP0000257083*) were more expressed in the 0.8ox sample (Figure 6).

An unbiased GO enrichment analysis revealed important processes that were selectively regulated by the different low oxygen levels applied to the harvested apples. GO terms overrepresented among genes that were more expressed in 0.4ox compared to 0.8ox samples (B vs. C, Table 2) included responses to biotic stimulus, defense response, and oxidation-reduction in the BP category. Although photosynthesis is not active in ripe harvested apples, genes associated with photosynthetic light reactions were more expressed in the 0.4ox sample and the GO term was over-represented. Our data indicate that storage in very low oxygen conditions, close to the compensation point, interferes with photosynthetic genes at different levels, such as PSII and PSI efficiency, ribulose-1,5-bisphosphate carboxylase/oxygenase (Rubisco) activity and photorespiration. Considering cellular components and molecular function, the membrane and oxido-reductase activity were over-represented GOs, respectively. It is interesting to note that three out of the four under-represented GO terms identified included translation (BP), ribosome (CC), and structural component of ribosome (MF).

**Table 2:** **GO enrichment analysis of genes more expressed in B (0.4 kPa oxygen) when compared with C (0.8 kPa oxygen) samples after 24 DIA**.

**GOcat**	**Ontology**	**Description**	**Size**	***p*-value**	**DEG**	**Enrichment**
GO:0009607	BP	Response to biotic stimulus	66	2.18E-24	22	OR
GO:0006952	BP	Defense response	79	1.97E-22	22	OR
GO:055114	BP	Oxidation-reduction process	1811	2.82E-14	71	OR
GO:0009765	BP	Photosynthesis, light harvesting	46	6.84E-14	13	OR
GO:0006826	BP	Iron ion transport	9	7.43E-08	5	OR
GO:0006879	BP	Cellular iron ion homeostasis	9	7.43E-08	5	OR
GO:0015936	BP	Coenzyme A metabolic process	23	7.25E-07	6	OR
GO:0006468	BP	Protein phosphorylation	1492	3.37E-06	45	OR
GO:0008299	BP	Isoprenoid biosynthetic process	30	3.92E-06	6	OR
GO:0006950	BP	Response to stress	98	0.000564	7	OR
GO:0006412	BP	Translation	923	1.24E-06	0	UR
GO:0016020	CC	Membrane	1232	0.000285	34	OR
GO:0005622	CC	Intracellular	1447	0.000324	7	UR
GO:0005840	CC	Ribosome	719	2.58E-05	0	UR
GO:0016491	MF	Oxidoreductase activity	1101	1.62E-11	48	OR
GO:0008199	MF	Ferric iron binding	9	7.43E-08	5	OR
GO:0005488	MF	Binding	64	3.50E-07	9	OR
GO:0004420	MF	Hydroxymethylglutaryl-CoA reductase (NADPH) activity	23	7.25E-07	6	OR
GO:0016705	MF	Oxidoreductase activity	207	2.23E-06	14	OR
GO:0004163	MF	Diphosphomevalonate decarboxylase activity	3	3.00E-06	3	OR
GO:0004672	MF	Protein kinase activity	1486	3.04E-06	45	OR
GO:0016706	MF	Oxidoreductase activity	137	5.33E-06	11	OR
GO:0005506	MF	Iron ion binding	266	9.06E-06	15	OR
GO:0016841	MF	Ammonia-lyase activity	12	1.95E-05	4	OR
GO:0020037	MF	Heme binding	303	4.13E-05	15	OR
GO:0009055	MF	Electron carrier activity	484	9.74E-05	19	OR
GO:0022857	MF	Transmembrane transporter activity	129	0.000114	9	OR
GO:0016760	MF	Cellulose synthase (UDP-forming) activity	42	0.000338	5	OR
GO:0016597	MF	Amino acid binding	44	0.000422	5	OR
GO:0004452	MF	Isopentenyl-diphosphate delta-isomerase activity	3	0.000619	2	OR
GO:0003735	MF	Structural constituent of ribosome	766	1.28E-05	0	UR

*GOseq Probability Weighting Function retrieve 27 over-represented (OR) and 4 under-represented (UR) GO terms among DEGs (qval < 0.001 and log_2_ FC>1)*.

The GO overrepresentation amongst genes more expressed at the higher (0.8 kPa) O_2_ level (Table 3) revealed considerably different GO terms such as transmembrane transport, sodium ion transport, and carbohydrate metabolic processes. Membrane, beta-galactosidase complex, and mitochondrial intermembrane space were overrepresented cellular components (CC). Transferase, transporter, and hydrolase activity were typically overrepresented molecular functions (MF).

**Table 3:** **GO enrichment analysis of genes more expressed in C (0.8 kPa oxygen) when compared with B (0.4 kPa oxygen) samples after 24 DIA**.

**GOcat**	**Ontology**	**Description**	**Size**	***p*-value**	**DEG**	**Enrichment**
GO:0055085	BP	Transmembrane transport	662	3.81E-11	28	OR
GO:0006814	BP	Sodium ion transport	38	5.98E-11	9	OR
GO:0005975	BP	Carbohydrate metabolic process	456	2.86E-05	15	OR
GO:0009790	BP	Embryo development	2	8.60E-05	2	OR
GO:0006013	BP	Mannose metabolic process	11	0.000124	3	OR
GO:0005991	BP	Trehalose metabolic process	3	0.000257	2	OR
GO:0006665	BP	Sphingolipid metabolic process	3	0.000257	2	OR
GO:0055114	BP	Oxidation-reduction process	1811	0.000418	32	OR
GO:0006825	BP	Copper ion transport	4	0.00051	2	OR
GO:0016020	CC	Membrane	1232	0.000102	26	OR
GO:0009341	CC	Beta-galactosidase complex	12	0.000164	3	OR
GO:0005758	CC	Mitochondrial intermembrane space	4	0.00051	2	OR
GO:0016021	CC	Integral component of membrane	1007	0.000547	21	OR
GO:0005622	CC	Intracellular	1447	0.000604	3	UR
GO:0016747	MF	Transferase activity, transferring acyl groups other than amino-acyl groups	172	2.48E-16	20	OR
GO:0005215	MF	Transporter activity	161	3.56E-11	15	OR
GO:0004553	MF	Hydrolase activity, hydrolyzing O-glycosyl compounds	256	5.76E-06	12	OR
GO:0000155	MF	Phosphorelay sensor kinase activity	26	9.31E-05	4	OR
GO:0015923	MF	Mannosidase activity	11	0.000124	3	OR
GO:0004565	MF	Beta-galactosidase activity	12	0.000164	3	OR
GO:0022857	MF	Transmembrane transporter activity	129	0.000213	7	OR
GO:0004348	MF	Glucosylceramidase activity	3	0.000257	2	OR
GO:0004555	MF	Alpha, alpha-trehalase activity	3	0.000257	2	OR
GO:0004559	MF	Alpha-mannosidase activity	14	0.000268	3	OR
GO:0016531	MF	Copper chaperone activity	4	0.00051	2	OR
GO:0016702	MF	Oxidoreductase activity	21	0.000933	3	OR
GO:0005515	MF	Protein binding	3238	0.000233	13	UR

*GOseq Probability Weighting Function retrieve 25 over-represented (OR) and 2 under-represented (UR) GO terms among DEGs (qval < 0.001 and log_2_ FC > 1)*.

Considering the whole set of DEGs when B vs. C samples were compared (Table S4), 2-oxoglutarate (2OG) and Fe (II)-dependent oxygenase (FeKGO) genes were highly affected by hypoxic conditions. One FeKGO gene (*MDP0000436890*) showed the highest FC (5.76) expression when comparing B vs. C samples and other nine FeKGO genes showed a strong upregulation (FC > 3) in 0.4ox samples. This thus, indicates that there is an intrinsic connection between oxygen deprivation and oxygenases in apples. Other genes with a very high expression in 0.4ox samples compared to 0.8ox were those encoding cytochrome P450 (12 out of 13 identified as DE), and 18 (out of 20 identified as DE) MLP-like proteins putatively involved in RNA processing/ribonucleases, defense and biotic stimulus responses. As far as the hormone signaling and metabolism is concerned, several auxin-related genes were more expressed in 0.4ox compared to 0.8ox samples, such as a SAUR-like auxin-responsive and an auxin-responsive family protein, three genes involved in brassinosteroid metabolism, two GAST1 protein homologs and one cytokinin oxidase. However, four DEGs involved in ethylene signal transduction were identified as being more expressed in the 0.8ox sample, as well as one gene putatively involved in cytokinin signal transduction.

In the 0.8ox sample, the gene showing the highest FC compared to the 0.4ox sample was a dicarboxylate/malate carrier gene (*MDP0000309741*, FC = 5.55), which might be related to a more pronounced catabolism of malic acid. Considering genes involved in transport, seven tonoplast dicarboxylate transporter and three sulfate transporter genes had higher expression in the 0.8ox sample with FC > 2. As reported above, transmembrane transport and specific ion transport are among the GO terms overrepresented in apples kept at 0.8ox compared to 0.4ox samples. This might indicate that intracellular trafficking in the apple cortex is highly sensitive to slightly different hypoxic conditions. Interestingly, nitrate reductase was also found to be more expressed in the 0.8ox sample compared to 0.4ox.

### Transcription factors and signal transduction components underlying changes in responses to different low O_2_ levels

Clearly, subtle changes in O_2_ levels lead to a very large shift in transcriptome composition (Figure 5). ERF TF abundance is important but only partially explains such changes since ERF-VIIs have not yet been reported to target such a large range of genes. To further highlight possible players in this relatively large transcriptomic change operating over a small O_2_ difference, differentially regulated TFs were identified.

A total of 63 putative TFs were differentially expressed (qth < 0.001 and |fth|> 1) of which, 18 were more expressed in 0.8ox and 45 more expressed in the 0.4ox sample (Table S7). In the 0.8ox sample, the TF showing the highest FC (4) was a member of the MADS box family (*MDP0000212925, AGAMOUS80*), whereas in the 0.4ox sample an AUX/IAA transcriptional regulator family protein (*MDP0000223496*) showed the highest FC (3.03), together with three other AUX/IAA genes. The lowest oxygen concentration induced an up-regulation of four WRKY, five HB, and two Zinc-finger genes.

A putative VQ motif gene (*MDP0000346969*) was found to be induced at 0.4 kPa, compared to 0.8 kPa O_2_. VQ proteins play an important role in plant growth, development, and response to environmental conditions, most likely by acting as cofactors of group I and IIc WRKY (Cheng et al., 2012). Two putative WRKY TFs (WRKY 23 *MDP0000652760* and WRKY33 *MDP0000708692*) were induced at 0.4 kPa oxygen compared to 0.8 kPa oxygen. These TFs belong to the WRKY group I (Rushton et al., 2010) and thereby could interact and co-operate with VQ proteins (Cheng et al., 2012). In addition, two other members of the WRKY TF family (*MDP0000134105, MDP0000127976*) were more expressed in the 0.4ox than 0.8ox sample.

## Discussion

It is well known that decreases in oxygen concentration as those used in CA protocols result in a retarded metabolism, reduced respiration, delayed ripening, and an extension of shelf life in several fruit species including apples (Yahia, 2009). We exploited an integrated transcriptomic and metabolic approach to characterize, as comprehensively as possible, apple cortex responses to different oxygen regimes adopted during apple storage. We found that in apple cortex, the overall metabolism is markedly affected throughout the imposed period of low oxygen stress (up to 60 days of storage in hypoxia) with responses that vary in terms of temporal pattern and intensity.

As observed in *Arabidopsis* (Blokhina et al., 2014), one of the main effects of hypoxic conditions in apple cortex is modulation of the oxygenase expression. In addition, the differential expression of a large number of members of the superfamily of cytochromes P450 may indicate the presence of finely-tuned mechanisms activating oxygen in signaling events (Lewis, 2002). GO classes related to important metabolic (e.g., carbohydrate) and biological (e.g., photosynthetic light reactions, transport, defense) processes and functions (e.g., hydrolase activity) were found to be highly affected by both hypoxic conditions. In addition, our data concerning the number of DEGs (Table S4) and the overall evaluation of the GO terms overrepresented when comparing 0.4 and 0.8ox samples (Tables 2, 3) indicate that the slightly different hypoxic conditions imposed have a profound effect on apple fruit physiology.

One of the main physiological effects of applying CA conditions to ripening fruit is the reduction in ethylene biosynthesis. Given the relationships between ethylene and ripening gene expression in climacteric fruits (Grierson, 2013), this impacts strongly on the ethylene-dependent processes occurring in detached climacteric fruit. In our trial, ethylene production was not measured, although Both et al. (2014) report that in Royal Gala apples, decreasing oxygen levels from 1 to 0.5 kPa lead to a reduction in ethylene biosynthesis after 8 months of storage and the following 6 days of shelf-life at 20°C. Considering the two main ethylene biosynthetic steps, ACC synthase and ACC oxidase, our data suggest that under hypoxic conditions ethylene production is negatively affected through a repressive effect on both *ACS* and *ACO* transcript accumulation, confirming previous results obtained by Gorny and Kader (1996). This repressive effect was more pronounced in 0.4ox and was removed by shifting 0.4–0.8 kPa at 31 DIA. The increasing levels of expression of both *ACS* and *ACO* genes observed in the Nox samples, was delayed in hypoxia in a concentration dependent way. There is thus a key difference between model plants subjected to hypoxia or flooding and detached apple fruit stored under low oxygen. In fact, although differences have been observed among species and organs, in whole plants the submergence adaptation is associated with ethylene production/accumulation (Steffen and Sauter, 2014).

### Slight changes in low oxygen concentration affect pyruvate metabolism, fermentative pathway, and amino acid accumulation

Changes in the pyruvate metabolism in apple fruit appear to be a key event occurring under low oxygen conditions, as observed in model species. The accumulation of ethanol under extremely low oxygen concentration and/or during prolonged CA storage has been reported (Mattheis et al., 1991) also in relation to the development of low oxygen-induced physiological disorders (Vandendriessche et al., 2013; Lumpkin et al., 2014). As observed by Lumpkin et al. (2014) in Red Delicious apples, the rate of ethanol accumulation increases with the decrease in O_2_ levels. The relationship between different low oxygen concentrations and ethanol accumulation was confirmed in our experimental conditions in which 0.4 kPa oxygen represented a significantly more intense stress condition compared to 0.8 kPa (Figures 2, 5). This subtle difference in terms of oxygen concentration is readily perceived and associated with the differential transcriptional up-regulation of *ADH* genes as early as 3 DIA (Figure 3) and after 24 DIA (Figure 6). However, post-transcriptional/post-translational regulation mechanisms modulating ethanolic fermentation cannot be ruled out (Bucher et al., 1994).

The differential effect of the two hypoxic levels on the fermentative pathway was confirmed by the observation that, after shifting the oxygen concentration from 0.4 to 0.8 kPa (at 30 DIA), the ethanol concentration at 60 DIA was not different in the two hypoxic samples (Figure 2). The different responses to the imposed conditions are even more evident when considering the changes occurring in terms of alanine concentration. The increase in alanine, one of the most salient effects of hypoxia (Limami, 2014), may also have an important role in apple fruit tissue, in maintaining the glycolytic flux by preventing excessive pyruvate accumulation, while retaining carbon resources within the cell (Rocha et al., 2010). Hatoum et al. (2014) reported increases in alanine concentrations in Braeburn apples after long-term storage (up to 8 months) under 2.5 kPa O_2_,/3.7 kPa CO_2_ conditions. In the same apple variety, Vandendriessche et al. (2013) showed different alanine accumulations in relation to a different composition of the atmosphere (changes in both oxygen and carbon dioxide concentrations) after 4 months storage. Under our experimental conditions, the increase in alanine content occurred very rapidly (3 DIA) in both hypoxic samples.

A comparison of gene expression patterns, reveals, in general, a correlation between the intensity of the hypoxic stress and the expression level (Figure 3), as also observed for specific hypoxia-related genes (e.g., pyruvate decarboxylase) in other fruit species such as *Citrus sinensis* (Pasentis et al., 2007) This is also confirmed by RNA-seq data showing that several ADH loci were more expressed in 0.4ox samples at 24 DIA. The accumulation of *AlaAT* transcripts was extremely abundant in 0.4ox samples, suggesting a low oxygen response threshold for this gene. This was confirmed by the effects on the specific transcript accumulation of shifting oxygen from 0.4 to 0.8 kPa (31 DIA), which is an extremely pronounced effect for *AlaAT*. In soybean roots, transcripts from *GmAlaAT* subfamily A increased during water logging, and declined to levels below the initial value during the recovery (re-oxygenation) treatment (Rocha et al., 2010). Taken together these data suggest that, in apple fruit, specific *AlaAT*s possess a high level of transcriptional control by factors modulated by slight changes in oxygen concentration. This also suggests that under hypoxic conditions the fate of pyruvate, as a substrate to produce acetaldehyde (ethanol), lactate, or alanine, is highly dependent on the different low oxygen concentrations. Gene expression data concerning *PDC, ADH, LDH*, and *AlaAT* indicate that, with the exception of *AlaAT* in 0.8ox samples, there was an up-regulation in response to hypoxia as early as after 3 DIA (Figure 3). All these genes belong to a core group of genes showing altered expression under hypoxia in model plant species (*Arabidopsis*, rice, and poplar, Mustroph et al., 2010) and, in apple fruit, their up-regulation appears to be transient with a general decrease in transcript accumulation in later stages of storage. Interestingly, among these genes, those that are homologous to core-responsive anaerobic genes in *Arabidopsis* (Mustroph et al., 2009) exhibited a peak of induction when fruit were kept at 0.4 kPa oxygen for 10 days, although they were significantly up-regulated in milder hypoxic conditions. *AlaAT*, instead, was only induced by the lowest oxygen concentration used in this study, suggesting it to be regulated by strict hypoxia- or anoxia-specific mechanism(s).

In addition to alanine, other aminoacids were affected in relation to the intensity of hypoxic stress (Table 1). Compared to the Nox sample, GABA, a well-known end-product of plant cells held under low oxygen conditions, showed an increasing trend (statistically significant only under 0.8ox conditions). Since no induction of glutamate decarboxylase (GAD) genes was observed under hypoxia, it can be hypothesized that post-transcription or post-translational regulation of this enzyme occurs in conditions of oxygen deficiency. Alternatively, either polyamine degradation or non-enzymatic proline oxidation could contribute to GABA accumulation under hypoxia (Shelp et al., 2012; Signorelli et al., 2015). In pears, increases in GABA content are the main markers of hypoxia (Pedreschi et al., 2009a): taken together, these findings may indicate that, as observed in other plant organs, also in fruit tissues GABA represents a key metabolite in the interface between C and N metabolism under energetically demanding stresses. Whether, as reported by Michaeli and Fromm (2015), GABA may also act a signaling molecule and/or a sensor for the energetic level of the fruit remains to be elucidatedInterestingly, GABA and alanine accumulation patterns showed a similar trend characterized by a transient increase in the early stages (3–17 DIA) of storage followed by a decrease. This suggests that in apple fruit the metabolisms of alanine and GABA are correlated. Asparagine and aspartate are significantly reduced by 0.4 but not by 0.8 kPa, which suggests that the two stress levels differently modulate the decrease in the carbon-flux into the TCA cycle. An accumulation of threonine accompanied by the attenuation of aspartate in 0.4ox samples could indicate that threonine synthesis is induced during extremely low oxygen stress, since the biosynthesis of threonine starts from aspartate. However, the synthesis of threonine requires energy, carbon, and nitrogen (Galili, 1995), and plants under oxidative stress tend to direct the metabolism toward saving ATP. Additional investigations are needed to elucidate this aspect of threonine metabolism under hypoxia. The general decrease of uridine in both hypoxic samples compared to the control (where uridine increases throughout the storage period) might be, as suggested by Loef et al. (1999), the consequence of the enhanced use, under the imposed stress, of preformed nucleotides, in order to avoid the high energy cost of a de-novo biosynthesis of uridine which represents an important cofactor in the use of sucrose. An attenuation of uridine has been detected in potatoes where oxygen concentration gradients are present within the growing tuber (Geigenberger et al., 2000).

### Carbohydrate, cell wall, and secondary metabolisms as well as intracellular transport processes are highly responsive to different hypoxic levels

In elongating plant organs, the decrease in ATP and the reduced level of energy production caused by hypoxia induce a number of reactions. The primary metabolism is affected in terms of the catabolic pathways that allow the hydrolysis of starch and the catabolism of sucrose. Based on our expression data, also this seems to occur in hypoxic detached apples.

The transient up-regulation of beta-amylase observed in Granny Smith apples stored under hypoxia can be considered as specific to organs accumulating starch (such as apples), since amylases are not up-regulated in *Arabidopsis* seedlings and poplar roots where starch reserve may be unavailable or not accessible (Mustroph et al., 2010). Apples accumulate starch during the late stages of development, and when stored (particularly for long periods), the starch content is still high. In starch-accumulating organs (such as coleoptiles and germinating rice seeds) starch degrading enzymes (α-amylase, β-amylase, debranching enzyme, α-glucosidase) are active and low oxygen conditions up-regulate amylase gene expression (in particular β-amylase) in several species (Mustroph et al., 2010). Also in apples, β-amylase appears to be a hypoxia-induced gene and might be involved in the decrease in starch content observed during CA storage (Gorin et al., 1978).

As reported in the vegetative tissues of several species, the prompt up-regulation of *SuSy* gene expression indicates that also in apple fruit, this is one of the core-responsive genes to hypoxia, which are highly sensitive to changes in oxygen concentration in the environment. The induction of *SuSy* has been associated with the activation of mechanisms that compensate for severe ATP deficiency by the induction of alternative pathways that use inorganic pyrophosphate (PPi) instead of ATP for phosphorylation reactions. Compared to the invertase-hexokinase pathway, *SuSy* activation is considered as a truly energy-saving pathway. Similarly to findings for *AlaAT*, there seems to be a strict relationship between the oxygen concentration and the level of *SuSy* gene expression induction in apple cortex.

To meet the energy demand in many plants subjected to hypoxic stress, an increase in carbohydrate flux through glycolysis occurs. One of the key steps in glycolysis is the conversion of fructose-6-phosphate to fructose-1,6-biphosphate catalyzed by PFK. In some plant species, PFK is induced under low oxygen conditions, and similarly to Susy, this is true for the form (PFP) that uses PPi instead of ATP in order to save energy (Bailey-Serres et al., 2012).

The rapid and marked up-regulation of the *PFK* expression indicates that also in apple fruit, as observed in sensitive and tolerant plants to hypoxia (Mustroph et al., 2013), PFK is highly sensitive to hypoxic conditions and is probably key in re-setting carbon metabolism under oxygen deficiency through an induction of the glycolytic pathway. Although *SuSy* and *PFK* resulted highly induced by hypoxic conditions, focusing on the first 60 days of low oxygen storage, our data do not show any significant differences among samples in terms of the main sugars (fructose, glucose, and sucrose) content (Table 1). This might be the result of post-transcriptional and post-translational regulatory mechanisms that are activated in relation to hypoxic stress. Mustroph et al. (2013) reported that while the genes were strongly expressed, the activities of PFK and PFP were only slightly increased in cell extracts of rice in response to short-term anoxic treatments. The same authors reported that different regulatory mechanisms concerning *PFK/PFP* gene expression and activity could be related to the different level of sensitivity/tolerance to low oxygen conditions. Whether this also occurs in apples still needs investigating.

RNA-seq data indicate that transmembrane carriers/ion transporters are among the GO terms overrepresented when comparing the two hypoxic conditions, and this suggests that ionic and osmotic homeostasis is altered under low oxygen stress in apple cortex with effects on cellular physiology and metabolism. The gene most expressed with the highest fold change in C (0.8ox) vs. B (0.4ox) sample is a dicarboxylate carrier. Regalado et al. (2013) characterized three *V. vinifera* mitochondrial dicarboxylate/tricarboxylate carriers (DTC) putatively involved in the transport of mitochondrial malate (and other organic acids). Two of these *DTC* genes showed a developmental expression pattern during grape berry ripening (when malic acid concentration decreases) suggesting they are involved in malic acid transport into mitochondria for oxidation. Since one of the main effects of lowering oxygen concentrations in DCA protocols is to maintain higher levels of organic acids (malic acid, as the main organic acid in apples), the higher expression of these dicarboxylate carrier/transporter genes in 0.8ox samples may be related to a more pronounced catabolism of malic acid.

Another effect of the low oxygen storage of apple fruit is the maintenance of flesh firmness, one of the most important quality parameters. In general, the lower the oxygen level the higher the values of apple flesh firmness both at the end of the storage period and during the post-storage life (Hennecke et al., 2008). The loss of flesh firmness is strictly related to changes in the composition, architecture, and physical properties of primary cell wall constituents. These changes are driven by specific enzymes (above all hydrolases) through reactions leading to solubilization and depolymerizations of two main domains: pectins and hemicellulose. In apples, the decrease in total pectin and hemicellulose content has been found to be higher under storage at 3% compared to 1% oxygen concentration where the free pectin content was lower (Siddiqui et al., 1996). Gwanpua et al. (2014) reported that a loss of neutral sugars, an increase in pectin solubility and a decrease in water soluble pectins are associated with Jonagold apple softening, which is more pronounced in fruit stored under normoxia than in CA. The key role played by pectin depolymerization process in apple softening has been clearly demonstrated in *PG1*-suppressed “Royal Gala” apples, which maintain higher firmness values after ripening (Atkinson et al., 2012). The lower expression in the 0.4ox sample of genes with important roles in pectin (polygalacturonases, pectate lyase) and hemicellulose/xyloglucan (β-xylosidase) depolymerization, together with one expansin and one xyloglucan endotransglysolyase (Figure 6) would explain, at the molecular level, the beneficial effects of extremely low oxygen concentrations on flesh firmness. Three pectinacetylesterase (PAE) genes were more expressed in the 0.4ox sample. No information is available in the literature concerning the possible role of PAE in the cell wall metabolism of ripening fruit and the biological function of pectin acetyl esterification is not clear. Renard and Jarvis (1999) suggested that de-acetylation favors pectin-calcium interactions resulting in strengthening the wall and middle lamella. In potato plants overexpressing PAE, the tuber cell wall was found to have greater failure strength and was stiffer, which was explained by increased interactions (ionic binding of divalent cations, hydrogen bond) between homogalacturonan monomers (Orfila et al., 2012). Perhaps this also occurred in apples stored at the lowest oxygen concentration used in our trial, and PAE may have a role, together with other cell wall enzymes, in altering the physical properties of primary cell wall in ripening fruit. Since in climacteric fruit, the up-regulation of some cell wall degrading enzymes at ripening is ethylene-dependent, it would be interesting to assess whether the mechanisms regulating the expression of these genes are directly related to hypoxic sensing mechanisms or to the reduction/inhibition of ethylene production and/or signal transduction.

With regard to specific secondary metabolism pathways, it seems that opposite effects are induced by varying the oxygen level on terpene synthesis and the earliest step of the phenylpropanoid pathway. In fact, a terpene synthase gene was more (FC > 2) expressed in the 0.8ox sample. This appears to be in agreement with findings of Both et al. (2014) who reported a decrease in a terpene compound in Royal Gala apples stored under decreasing levels of oxygen concentration (from 1.0 to 0.5 kPa). Phenylalanine ammonia lyase genes (PAL) were more expressed (two at FC > 2 and two at FC > 1) under the lowest oxygen concentrations. The production of different classes of phenol compounds through the activation of the phenylpropanoid pathway and the up-regulation of *PAL* expression has been associated with the responses of detached fruit to various kinds of abiotic stress such as cold (Lo Piero et al., 2005) and wounding (Tosetti et al., 2014). Based on the RNA-seq data, it can be hypothesized that the earliest step in this secondary metabolic pathway is also highly sensitive to different levels of hypoxic stresses. Since the expression of a cinnamoyl CoA reductase was also induced, the production of defense-related metabolites, including lignin, may possibly be induced under extreme stress conditions. Mellidou et al. (2014) reported an up-regulation of a cinnamoyl reductase gene in Braeburn apples affected by the CA-induced browning disorder.

### Transcription factors are selectively modulated in hypoxic apples suggesting the presence of finely-tuned regulatory mechanisms

The high number of TFs that were differentially expressed in the 0.4ox vs. 0.8ox sample comparison clearly indicates that the responses to different levels of hypoxia are (also) the result of a modulation of signaling events leading to differentially regulated transcriptional activity, which is apparently more pronounced when the stress is more intense (45 and 18 putative TFs were more expressed in 0.4ox and 0.8ox samples, respectively). In harvested fruit, a modulation of WRKY TFs has been associated with different responses/tolerance to stresses such as wounding (Tosetti et al., 2014) and low temperature (Sanchez-Ballesta et al., 2003).

The involvement of AP2/ERFs members in the responses to different stresses including oxygen deprivation was demonstrated by Nakano et al. (2006) and then by Licausi et al. (2010) who described two members of the group VII ERF proteins (HRE1 and HRE2) as possible regulators of the hypoxic response in *Arabidopsis*. The transcription of these two genes is highly up-regulated under low oxygen conditions, which is also what we observed in apple cortex where the two orthologues of *Arabidopsis* HRE (*MDP0000288465* and *MDP0000848905*) showed a prompt up-regulation under hypoxic conditions, with the highest levels of transcript accumulation occurring in the 0.4ox samples. It is still not clear whether the induced expression of these two TFs in apple is required to maintain the expression of fermentative and other hypoxic genes to ameliorate tissue survival under low oxygen condition as observed in *Arabidospis* (Licausi et al., 2011b). The other members of the *Arabidopsis* group VII ERF subfamily, which are constitutively expressed, have been proposed as candidates for early oxygen sensing through the N-end rule pathway (NERP; Bui et al., 2015).

The results of western blot analysis clearly indicate that the apple ERF-VII MdRAP2.12 protein differentially accumulates in samples held at different oxygen concentrations, with the highest level reached in samples maintained at the lowest oxygen concentrations, whereas under normoxia no accumulation of the protein was detected. The different accumulation of MdRAP2.12 protein in the apple cortex after 24 DIA indicates a strong inhibition of the plant cysteine oxidase activity, which targets ERF-VII proteins toward proteolysis, at 0.4 KPa O_2_ in the surrounding environment (Weits et al., 2014). Taken together these results suggest that the NERP involving the degradation of RAP2.12 and the oxygen sensing mechanisms described in *Arabidopsis* are also present in apple fruit cortex tissues. The rapid and marked up-regulation of HRE genes also seems to indicate that these TFs are the elements primarily responding to changes in oxygen concentration possibly sensed by the levels of RAP2.12 protein, and responsible for the regulation of the expression of hypoxia-induced genes (e.g., *ADH*, Licausi et al., 2011a).

Apple varieties react differently to extremely low oxygen conditions during storage, in particular in terms of ethanol production and accumulation, as observed, for example, in Red Delicious fruit which accumulate much more ethanol than Granny Smith (Zanella and Tonutti, unpublished). If this is the result of different oxygen sensing mechanisms involving group VII ERF TFs in general and in particular RAP2.12 remains to be elucidated.

## Conclusions

Harvested apple fruit maintained under extreme low oxygen conditions for up to 60 days undergo marked changes in their overall metabolism. Based on our integrated metabolic and transcriptomic approach, some processes appear to be already affected at the earliest stages of exposure to hypoxia. It can thus be concluded that:

- Apple fruit cortex tissues are highly sensitive even to subtle differences in oxygen concentrations close to the anaerobic compensation point. This is reflected in the ethanol concentration, the expression of hypoxia marker genes such as *AlaAT* and *SuSy*, and the large number of RNA-seq identified genes, which are extremely reactive to small changes in oxygen concentration;
- The oxygen sensing mechanism based on the N-end rule pathway and on the post-translational regulation of group VII ERF protein stability seems also to be present and active in apple fruit. RAP2.12 action is the likely mechanism by which subtle changes take place in responses to different hypoxic conditions;
- Some hypoxia responsive metabolic processes appear to be shared with those of vegetative tissues of model species, others (e.g., cell wall, organic acid) appear to be specific to fruit tissues. The observed metabolic reset occurring in apple cortex under hypoxic conditions appears to be the result of regulatory mechanisms modulating not only gene expression, but also post-transcriptional and post-translational processes;
- Specific molecular and metabolic changes occur at the earliest stages of the imposed stress conditions and some of them appear to be transient. Whether some of these changes represent a sort of a rapid adaptation response to the stress remains to be elucidated.

## Author contributions

BR and PT designed the experimental trials; MZ, BR performed the experiments and collected the samples; SB, CL, CS, LT carried out the NMR analyses; DC, MZ performed the gene expression and western blot analyses, respectively; DC, AC, HV, AZ carried out the bioinformatic analyses; SB, DC, FL, BR, HV, PT analyzed the data and discussed the results; DC, SB, BR, and PT wrote the article. All authors read and approved the final manuscript.

## Funding

This work was financially supported by Marvil Engineering s.r.l., 39040 Magré s.s.d.v. (Bolzano, Italy), by Progetto AGER Melo, grant n° 2010-2119, and by grants from Scuola Superiore Sant'Anna (Ricerca d'Ateneo) to P.T. and from University of Padova (Ricerca scientifica Ex-60%) to B.R. M.Z. was supported by the University of Padova (Assegno di Ricerca) and L.T. by Fondazione Veronesi through a Post-Doctoral Fellowship-2015.

### Conflict of interest statement

The authors declare that the research was conducted in the absence of any commercial or financial relationships that could be construed as a potential conflict of interest.

## Abbreviations

β-amy: β-amylase
ACS: ACC-synthase
ACO: ACC oxidase
ADH: alcohol dehydrogenase
AlaAT: alanine aminotransferase
CA: controlled atmosphere
CHO: carbohydrate
DCA: dynamic CA
DEGs: differentially expressed genes
DIA: days in atmospheres
ERFs: ethylene responsive factors
GABA: γ-aminobutirate
GO: gene ontology
LDH: lactate dehydrogenase
NERP: N-end rule pathway
Nox: normoxic conditions
PDC: pyruvate decarboxylase
PFK: phosphofructokinase
SuSy: sucrose synthase
TFs: transcription factors.
