# Supplementary material for: Extreme Hypoxic Conditions Induce Selective Molecular Responses and Metabolic Reset in Detached Apple Fruit
Source: Front Plant Sci. 2016 Feb 16;7:146. doi: 10.3389/fpls.2016.00146 (PMC4754620; doi:10.3389/fpls.2016.00146)
Supplement: Supplementary file 1 [file Table1.DOCX]

**Cukrov et al., supplementary material. Table S1** Primer sequences for RT-qPCR.

| **Gene** | **Forward primer (5’-3’)** | **Reverse primer (5’-3’)** |
| --- | --- | --- |
| *PYRUVATE DECARBOXYLASE*  *MDP0000800945* | CAAGGCAGTAAAGCCGGTTA | AAATCGGTCCAGCAAACAAG |
| *SUCROSE SYNTHASE*  *MDP0000250070* | TCCGTGTTCACTGCTACGAG | GCCTCAAGAAGGTCCAACAG |
|  |  |  |
| *PHOSPHOFRUCTOKINASE*  *MDP0000131308* | AGTCGTGGAGTGGTGGAATC | TAGAGGGTGAGGGCTTCAGA |
|  |  |  |
| *ACC SYNTHASE*  *MDP0000413933*   \|  \|  \| \| --- \| --- \| | AAGTGGCGAACTGGAGTCGA | GGTTTGATGGGTTCGTGACC |
| *ACC OXIDASE*  *MDP0000195885* | CAGTCGGATGGGACCAGAA | GCTTGGAATTTCAGGCCAGA |
| *ALCOHOL DEHYDROGENASE MDP0000594290*  *ERF* | GGAAGCACTGAAGCCATGAT | CTCCACGACAGAGGGAATGT |
| *MDP0000848905* | CGGTGGTGCTATAATCTCCG | GGAATTGAGTCGGTGTGAGTAGTT |
| *MDP0000288465* | CTCCCTTCGCCAAGTTCG | TTGAGTCGGTGCGATTAACC |
| *MDP0000413387* | CCAGAAGCCCAAACCATCAG | TTCCTCGGCGGTGTTGTA |
| *MDP0000128979* | GGTGGGGAAATGTATGCTAAGA | GTCATCCAGCATCCACAGG |
| *MDP0000308922* | CTTCTGCAAAGCGTTCTGTG | GGCAGGATCGGATGGAG |
| *MDP0000403580* | TTCTGCAAAGCGTTCCATC | TTCATTGGCAGGGAAGGTG |
|  |  |  |
| *ALANINE*  *AMINOTRANSFEREASE*  *MDP0000319263* | TGCTGTCCGAGGTGAAATCGTC | AGCCCGGATTCGCCTTTAACTC |
| *BETA-AMYLASE 6*  *MDP0000764433*  *MDP0000142134* | CTATGTGCCGATCTTCGTGA | ACTGCTTGAAACACGCTCCT |
| *LACTATE DEHYDROGENASE*  *MDP0000568449*  *MDP0000143956* | CATAAAACTCCTTCAGGCTCCA | GTGSGGTCTTGGGTGAGGAT |
| *HOUSEKEEPING GENE*  *MDP0000375455* | CTCGTCGTCTTGTTGTTCCCTGA | GCCTAAGGACAGGTGGTCTATG |
|  |  |  |
