# Supplementary material for: Extreme Hypoxic Conditions Induce Selective Molecular Responses and Metabolic Reset in Detached Apple Fruit
Source: Front Plant Sci. 2016 Feb 16;7:146. doi: 10.3389/fpls.2016.00146 (PMC4754620; doi:10.3389/fpls.2016.00146)
Supplement: Supplementary file 2 [file Table2.DOCX]

**Cukrov et al. supplementary material. Table S2** Correspondence between MDP, GeneBank Accession and gi NCBI numbers.

| **MDP indentification** | **GeneBank Accession** | **gi identification** |
| --- | --- | --- |
| MDP0000248863 | XM_008375387.1 | 657963976 |
| MDP0000248942 | XM_008369110.1 | 657943362 |
| MDP0000256797 | XM_008379290.1 | 657971451 |
| MDP0000305455 | XM_008370546.1 | 657943395 |
| MDP0000267885 | XM_008365212.1 | 658055375 |
| MDP0000551876 | XM_008349135.1 | 658023912 |
| MDP0000499668 | XM_008352987.1 | 658031476 |
| MDP0000129112 | XM_008340871.1 | 658007813 |
| MDP0000170286 | XM_008345215.1 | 658016191 |
| MDP0000321380 | XM_008341454.1 | 658008957 |
| MDP0000127640 | XM_008365212.1 | 658055375 |
| MDP0000429824 | XM_008389108.1 | 657990208 |
| MDP0000233401 | XM_008345215.1 | 658016191 |
| MDP0000881409 | XM_008343854.1 | 658013553 |
| MDP0000319266 | XM_008367089.1 | 658058997 |
| MDP0000155345 | XM_008391653.1 | 657995109 |
| MDP0000250876 | XM_008350792.1 | 658027168 |
| MDP0000736852 | XM_008375976.1 | 657965107 |
| MDP0000243086 | XM_008377010.1 | 657967084 |
| MDP0000229344 | XM_008386440.1 | 657985118 |
| MDP0000288465 | XM_008388440.1 | 657988952 |
| MDP0000205358 | XR_529192.1 | 657957661 |
| MDP0000284467 | XM_008367723.1 | 658060221 |
| MDP0000264347 | XM_008386719.1 | 657985696 |
| MDP0000256486 | XM_008340248.1 | 657948584 |
| MDP0000564897 | XM_008340338.1 | 658006782 |
| MDP0000933110 | XM_008380355.1 | 657973545 |
| MDP0000141097 | XM_008339955.1 | 658006048 |
| MDP0000575908 | XM_008339874.1 | 658005888 |
| MDP0000268306 | XM_008373423.1 | 657960153 |
| MDP0000539118 | XM_008353007.1 | 658031511 |
| MDP0000708692 | XM_008371989.1 | 657957422 |
| MDP0000809773 | XM_008382344.1 | 657977317 |
| MDP0000841002 | XM_008379711.1 | 657972261 |
| MDP0000127976 | XM_008383213.1 | 657978983 |
| MDP0000138052 | XM_008377832.1 | 657968701 |
| MDP0000683814 | XM_008340392.1 | 658006882 |
| MDP0000237396 | XM_008379514.1 | 657945032 |
| MDP0000211459 | XM_008358912.1 | 658043011 |
| MDP0000652760 | XM_008359960.1 | 658045023 |
| MDP0000209313 | XM_008372463.1 | 657958329 |
| MDP0000134105 | XM_008359960.1 | 658045023 |
| MDP0000253285 | XM_008349260.1 | 658024153 |
| MDP0000258562 | XM_008379796.1 | 657972441 |
| MDP0000220601 | XM_008351116.1 | 657950716 |
| MDP0000135540 | XM_008388997.1 | 657989995 |
| MDP0000248279 | XM_008379055.1 | 657971015 |
| MDP0000238683 | XM_008367727.1 | 658060229 |
| MDP0000185616 | XM_008354701.1 | 658034805 |
| MDP0000318244 | XM_008365257.1 | 658055467 |
| MDP0000699761 | XM_008353007.1 | 658031511 |
| MDP0000218986 | XM_008365257.1 | 658055467 |
| MDP0000319456 | XR_527852.1 | 658051176 |
| MDP0000167107 | XM_008376540.1 | 657966158 |
| MDP0000212925 | XM_008365794.1 | 658056520 |
| MDP0000123816 | XM_008349260.1 | 103410576 |
| MDP0000202548 | XM_008392009.1 | 103452508 |
| MDP0000249034 | XM_008346056 | 103407105 |
| MDP0000270789 | XR_524203.1 | 103403600 |
| MDP0000320719 | XM_008371579.1 | 103433333 |
| MDP0000653903 | XM_008390196.1 | 103450804 |
| MDP0000223496 | XM_008349260.1 | 658024153 |
