# Supplementary material for: Extreme Hypoxic Conditions Induce Selective Molecular Responses and Metabolic Reset in Detached Apple Fruit
Source: Front Plant Sci. 2016 Feb 16;7:146. doi: 10.3389/fpls.2016.00146 (PMC4754620; doi:10.3389/fpls.2016.00146)
Supplement: Supplementary file 3 [file Table3.DOCX]

**Cukrov et al. supplementary material**

**Table S3** cDNA libraries of Granny Smith cortex tissue.

| Library | Reads used by Top_hat | Mapped | Unmapped | % of mapped reads |
| --- | --- | --- | --- | --- |
| 0.4ox_replicate 1 | 10327606 | 9125948 | 1201658 | 88.36 |
| 0.4ox_replicate 2 | 6926279 | 6123146 | 803133 | 88.40 |
| 0.4ox_replicate 3 | 10925992 | 9684217 | 1241775 | 88.63 |
| 0.8ox_replicate 1 | 16482537 | 14592530 | 1890007 | 88.53 |
| 0.8ox_replicate 2 | 7036035 | 6229908 | 806127 | 88.54 |
| 0.8ox_replicate 3 | 11364947 | 10010728 | 1354219 | 88.08 |
| T0_replicate 1 | 43747406 | 38925955 | 4821451 | 88.98 |
| T0_replicate 2 | 10465461 | 9302533 | 1162928 | 88.89 |
| T0_replicate 3 | 9170374 | 8144746 | 1025628 | 88.82 |
