# Supplementary material for: Extreme Hypoxic Conditions Induce Selective Molecular Responses and Metabolic Reset in Detached Apple Fruit
Source: Front Plant Sci. 2016 Feb 16;7:146. doi: 10.3389/fpls.2016.00146 (PMC4754620; doi:10.3389/fpls.2016.00146)
Supplement: Supplementary file 5 [file Table5.DOCX]

**Cukrov et al., supplementary material. Table S5.** Genes up-regulated in both hypoxic samples compared to T0: GO over-represented terms.

| GOcat | Ontology | Description | Size | p-value | DEG # |
| --- | --- | --- | --- | --- | --- |
| GO:0009607 | BP | response to biotic stimulus | 66 | 5.41E-12 | 16 |
| GO:0055114 | BP | oxidation-reduction process | 1811 | 9.11E-12 | 95 |
| GO:0042398 | BP | cellular modified amino acid biosynthetic process | 26 | 1.22E-11 | 11 |
| GO:0006952 | BP | defense response | 79 | 1.01E-10 | 16 |
| GO:0006096 | BP | glycolytic process | 115 | 5.99E-10 | 18 |
| GO:0005985 | BP | sucrose metabolic process | 16 | 5.58E-08 | 7 |
| GO:0006468 | BP | protein phosphorylation | 1492 | 1.68E-06 | 68 |
| GO:0006144 | BP | purine nucleobase metabolic process | 5 | 1.90E-06 | 4 |
| GO:0006355 | BP | regulation of transcription, DNA-templated | 1101 | 1.09E-05 | 52 |
| GO:0038032 | BP | termination of G-protein coupled receptor signaling pathway | 3 | 1.55E-05 | 3 |
| GO:0006094 | BP | gluconeogenesis | 24 | 0.000275632 | 5 |
| GO:0006222 | BP | UMP biosynthetic process | 6 | 0.00029379 | 3 |
| GO:0006950 | BP | response to stress | 98 | 0.000790032 | 9 |
| GO:0000160 | BP | phosphorelay signal transduction system | 63 | 0.000974022 | 7 |
| GO:0016872 | MF | intramolecular lyase activity | 26 | 1.22E-11 | 11 |
| GO:0047800 | MF | cysteamine dioxygenase activity | 13 | 3.53E-07 | 6 |
| GO:0016706 | MF | oxidoreductase activity | 137 | 3.59E-07 | 16 |
| GO:0005506 | MF | iron ion binding | 266 | 1.14E-06 | 22 |
| GO:0004672 | MF | protein kinase activity | 1486 | 1.47E-06 | 68 |
| GO:0004347 | MF | glucose-6-phosphate isomerase activity | 10 | 2.19E-06 | 5 |
| GO:0016491 | MF | oxidoreductase activity | 1101 | 2.51E-06 | 54 |
| GO:0000287 | MF | magnesium ion binding | 128 | 4.18E-06 | 14 |
| GO:0030976 | MF | thiamine pyrophosphate binding | 36 | 2.63E-05 | 7 |
| GO:0004743 | MF | pyruvate kinase activity | 32 | 0.000124376 | 6 |
| GO:0030955 | MF | potassium ion binding | 32 | 0.000124376 | 6 |
| GO:0016705 | MF | oxidoreductase activity, acting on paired donors, with incorporation or reduction of molecular oxygen | 207 | 0.000242892 | 15 |
| GO:0020037 | MF | heme binding | 303 | 0.000255163 | 19 |
| GO:0004152 | MF | dihydroorotate dehydrogenase activity | 6 | 0.00029379 | 3 |
| GO:0000155 | MF | phosphorelay sensor kinase activity | 26 | 0.000409291 | 5 |
| GO:0004332 | MF | fructose-bisphosphate aldolase activity | 15 | 0.000423956 | 4 |
| GO:0000156 | MF | phosphorelay response regulator activity | 56 | 0.000474657 | 7 |
| GO:0009055 | MF | electron carrier activity | 484 | 0.000584247 | 25 |
| GO:0005524 | MF | ATP binding | 3104 | 0.0009504 | 105 |
