# Supplementary material for: Extreme Hypoxic Conditions Induce Selective Molecular Responses and Metabolic Reset in Detached Apple Fruit
Source: Front Plant Sci. 2016 Feb 16;7:146. doi: 10.3389/fpls.2016.00146 (PMC4754620; doi:10.3389/fpls.2016.00146)
Supplement: Supplementary file 6 [file Table6.DOCX]

**Cukrov et al., supplementary material. Table S6.** Genes down-regulated in both hypoxic samples compared to T0: GO over-represented terms.

| GOcat | Onthology | Description | Size | p-value | DEG# |
| --- | --- | --- | --- | --- | --- |
| GO:0055114 | BP | oxidation-reduction process | 1811 | 2.19E-21 | 167 |
| GO:0044237 | BP | cellular metabolic process | 187 | 1.74E-11 | 32 |
| GO:0008152 | BP | metabolic process | 1215 | 5.18E-11 | 102 |
| GO:0006694 | BP | steroid biosynthetic process | 138 | 8.03E-10 | 25 |
| GO:0005975 | BP | carbohydrate metabolic process | 456 | 3.16E-09 | 49 |
| GO:0055085 | BP | transmembrane transport | 662 | 1.52E-08 | 61 |
| GO:0007585 | BP | respiratory gaseous exchange | 7 | 3.90E-08 | 6 |
| GO:0051258 | BP | protein polymerization | 48 | 6.60E-08 | 13 |
| GO:0007010 | BP | cytoskeleton organization | 19 | 5.12E-07 | 8 |
| GO:0045226 | BP | extracellular polysaccharide biosynthetic process | 42 | 9.83E-07 | 11 |
| GO:0009082 | BP | branched-chain amino acid biosynthetic process | 17 | 3.26E-06 | 7 |
| GO:0008654 | BP | phospholipid biosynthetic process | 14 | 1.29E-05 | 6 |
| GO:0046373 | BP | L-arabinose metabolic process | 6 | 4.52E-05 | 4 |
| GO:0019673 | BP | GDP-mannose metabolic process | 3 | 7.62E-05 | 3 |
| GO:0009073 | BP | aromatic amino acid family biosynthetic process | 20 | 0.000134 | 6 |
| GO:0006544 | BP | glycine metabolic process | 31 | 0.000262 | 7 |
| GO:0006563 | BP | L-serine metabolic process | 31 | 0.000262 | 7 |
| GO:0008652 | BP | cellular amino acid biosynthetic process | 41 | 0.000282 | 8 |
| GO:0006184 | BP | GTP catabolic process | 32 | 0.000323 | 7 |
| GO:0006012 | BP | galactose metabolic process | 16 | 0.000403 | 5 |
| GO:0015979 | BP | photosynthesis | 105 | 0.000509 | 13 |
| GO:0030036 | BP | actin cytoskeleton organization | 25 | 0.00051 | 6 |
| GO:0043234 | CC | protein complex | 48 | 6.60E-08 | 13 |
| GO:0016020 | CC | membrane | 1232 | 2.41E-07 | 91 |
| GO:0015629 | CC | actin cytoskeleton | 13 | 7.66E-06 | 6 |
| GO:0005740 | CC | mitochondrial envelope | 16 | 3.20E-05 | 6 |
| GO:0009317 | CC | acetyl-CoA carboxylase complex | 3 | 7.62E-05 | 3 |
| GO:0005874 | CC | microtubule | 47 | 0.000138 | 9 |
| GO:0016021 | CC | integral component of membrane | 1007 | 0.000213 | 67 |
| GO:0016491 | MF | oxidoreductase activity | 1101 | 9.03E-17 | 110 |
| GO:0050662 | MF | coenzyme binding | 201 | 1.22E-10 | 32 |
| GO:0003824 | MF | catalytic activity | 963 | 2.19E-09 | 82 |
| GO:0016616 | MF | oxidoreductase activity, acting on the CH-OH group of donors, NAD or NADP as acceptor | 242 | 3.75E-09 | 33 |
| GO:0003854 | MF | 3-beta-hydroxy-delta5-steroid dehydrogenase activity | 130 | 1.39E-07 | 21 |
| GO:0008831 | MF | dTDP-4-dehydrorhamnose reductase activity | 42 | 9.83E-07 | 11 |
| GO:0022857 | MF | transmembrane transporter activity | 129 | 2.28E-06 | 19 |
| GO:0004329 | MF | formate-tetrahydrofolate ligase activity | 4 | 3.23E-06 | 4 |
| GO:0003849 | MF | 3-deoxy-7-phosphoheptulonate synthase activity | 13 | 7.66E-06 | 6 |
| GO:0004455 | MF | ketol-acid reductoisomerase activity | 20 | 1.16E-05 | 7 |
| GO:0046556 | MF | alpha-L-arabinofuranosidase activity | 6 | 4.52E-05 | 4 |
| GO:0008446 | MF | GDP-mannose 4,6-dehydratase activity | 3 | 7.62E-05 | 3 |
| GO:0004553 | MF | hydrolase activity, hydrolyzing O-glycosyl compounds | 256 | 0.000103 | 25 |
| GO:0008134 | MF | transcription factor binding | 13 | 0.000132 | 5 |
| GO:0050660 | MF | flavin adenine dinucleotide binding | 124 | 0.000252 | 15 |
| GO:0004372 | MF | glycine hydroxymethyltransferase activity | 31 | 0.000262 | 7 |
| GO:0003978 | MF | UDP-glucose 4-epimerase activity | 15 | 0.000287 | 5 |
| GO:0016780 | MF | phosphotransferase activity, for other substituted phosphate groups | 9 | 0.000342 | 4 |
| GO:0048037 | MF | cofactor binding | 45 | 0.000547 | 8 |
| GO:0005507 | MF | copper ion binding | 95 | 0.000688 | 12 |
| GO:0004806 | MF | triglyceride lipase activity | 49 | 0.000985 | 8 |
