# Supplementary material for: Extreme Hypoxic Conditions Induce Selective Molecular Responses and Metabolic Reset in Detached Apple Fruit
Source: Front Plant Sci. 2016 Feb 16;7:146. doi: 10.3389/fpls.2016.00146 (PMC4754620; doi:10.3389/fpls.2016.00146)
Supplement: Supplementary file 7 [file Table7.DOCX]

**Cukrov et al., supplementary material. Table S7.** Transcription factors differentially expressed after 24 DIA in C (0.8 kPa ox) vs B (0.4 kPa ox) samples

| Gene ID | *Arabidopsis* ortholog | logFC  (0.8ox/0.4ox) | Description | GOcat | TF family |  |  |
| --- | --- | --- | --- | --- | --- | --- | --- |
| *MDP0000267885* | *AT4G32010.1* | 1.5671 | HSI2-like 1 | GO:0003677,GO:0006355 | ABI3/VP1-related B3-domain-containing |  |  |
| *MDP0000127640* | *AT4G32010.1* | 1.4879 | HSI2-like 1 | GO:0003677, GO:0006355,GO:0008270 |  |  |  |
| *MDP0000229344* | *AT2G30470.1* | 1.4303 | high-level expression of sugar-inducible gene 2 | GO:0003677, GO:0006355,GO:0008270 |  |  |  |
| *MDP0000683814* | *AT4G39780.1* | 1.7353 | Integrase-type DNA-binding superfamily protein | GO:0003700,GO:0006355 | AP2/EREBP, APETALA2/  EREB protein family |  |  |
| *MDP0000258562* | *AT2G23340.1* | -1.1158 | DREB and EAR motif protein 3 | GO:0003700,GO:0006355 |  |  |  |
| *MDP0000288465* | *AT2G47520.1* | -1.1566 | Integrase-type DNA-binding superfamily protein | GO:0003700,GO:0006355 |  |  |  |
| *MDP0000211459* | *AT1G19850.1* | 1.4654 | Transcriptional factor B3 family protein / auxin-responsive factor AUX/IAA-related | GO:0006355,GO:0005634, GO:0003677,GO:0009725 | ARF |  |  |
| *MDP0000268306* | *AT1G19220.1* | -1.3243 | auxin response factor 19 | GO:0006355,GO:0005634, GO:0003677,GO:0009725 |  |  |  |
| *MDP0000218986* | *AT2G28500.1* | 1.5894 | LOB domain-containing protein 11 | unknown | AS2 |  |  |
| *MDP0000318244* | *AT2G28500.1* | 1.5894 | LOB domain-containing protein 11 | unknown |  |  |  |
| *MDP0000881409* | *AT3G02550.1* | -1.1212 | LOB domain-containing protein 41 | unknown |  |  |  |
| *MDP0000155345* | *AT1G67310.1* | -1.4326 | Calmodulin-binding transcription activator protein with CG-1 and Ankyrin domains | GO:0005515 | AtSR |  |  |
| *MDP0000284467* | *AT2G33310.2* | 1.4913 | auxin-induced protein 13 | GO:0006355,GO:0005634 | Aux/IAA family | | |
| *MDP0000250876* | *AT4G28640.3* | 1.3082 | indole-3-acetic acid inducible 11 | GO:0006355,GO:0005634 |  | |  |
| *MDP0000123816* | *AT1G04250.1* | -1.0851 | AUX/IAA transcriptional regulator family protein | GO:0006355,GO:0005634 |  | |  |
| *MDP0000253285* | *AT1G04250.1* | -1.0986 | AUX/IAA transcriptional regulator family protein | GO:0006355,GO:0005634 |  | |  |
| *MDP0000270789* | *AT2G33310.1* | -1.9120 | auxin-induced protein 13 | GO:0006355,GO:0005634 |  | |  |
| *MDP0000223496* | *AT1G04250.1* | -3.0389 | AUX/IAA transcriptional regulator family protein | GO:0006355,GO:0005634 |  | |  |
| *MDP0000205358* | *AT1G09530.1* | 1.0778 | phytochrome interacting factor 3 | GO:0030528,GO:0006355 | bHLH | |  |
| *MDP0000499668* | *AT2G30000.1* | 1.3574 | PHF5-like protein | unknown | bZIP |  |  |
| *MDP0000138052* | *AT2G46270.2* | 1.2689 | G-box binding factor 3 | GO:0003700,GO:0043565,  GO:0046983,GO:0006355,  GO:0003677,GO:0006351,  GO:0005634 |  |  |  |
| *MDP0000129112* | *AT5G44080.1* | -1.6361 | Basic-leucine zipper (bZIP) transcription factor family protein | GO:0003700,GO:0043565,  GO:0046983,GO:0006355 |  |  |  |
| *MDP0000232445* | *AT1G06040.1* | -1.1246 | B-box zinc finger family protein | GO:0008270,GO:0005622 | C2C2(Zn) CO-like, Constans-like zinc finger family |  |  |
| *MDP0000321380* | *AT1G68520.1* | -1.1821 | B-box type zinc finger protein with CCT domain | GO:0008270,GO:0005622,  GO:0005515 |  |  |  |
| *MDP0000185616* | *AT1G68520.1* | -1.2996 | B-box type zinc finger protein with CCT domain | GO:0008270,GO:0005622,  GO:0005515 |  |  |  |
| *MDP0000551876* | *AT1G75540.1* | -1.5531 | salt tolerance homolog2 | GO:0008270,GO:0005622 |  |  |  |
| *MDP0000170286* | *AT4G27810.1* | -1.6622 | unknown | unknown | C2C2(Zn) DOF zinc finger family |  |  |
| *MDP0000233401* | *AT4G27810.1* | -1.7384 | unknown | unknown |  |  |  |
| *MDP0000248942* | *AT3G54810.1* | -1.2378 | Plant-specific GATA-type zinc finger transcription factor family protein | GO:0003700,GO:0008270,  GO:0043565,GO:0006355 | C2C2(Zn) GATA |  |  |
| *MDP0000841002* | *AT4G29190.1* | -1.1273 | Zinc finger C-x8-C-x5-C-x3-H type family protein | GO:0003676,GO:0008270 | C3H zinc finger family |  |  |
| *MDP0000320719* | *AT2G37060.1* | 1.7811 | nuclear factor Y, subunit B8 | GO:0043565,GO:0005622,  GO:0003677 | CCAAT box binding factor family, HAP3 |  |  |
| *MDP0000539118* | *AT1G08970.1* | -1.3853 | nuclear factor Y, subunit C9 | GO:0003677,GO:0043565,  GO:0005622 | CCAAT box binding factor family, HAP5 |  |  |
| *MDP0000699761* | *AT1G08970.1* | -1.3853 | nuclear factor Y, subunit C9 | GO:0003677,GO:0043565,  GO:0005622 |  |  |  |
| *MDP0000248863* | *AT4G18020.1* | -1.6654 | CheY-like two-component responsive regulator family protein | GO:0000156,GO:0000160,  GO:0006355,GO:0003677 | G2-like transcription factor family, GARP |  |  |
| *MDP0000809773* | *AT3G24120.1* | -1.8080 | Homeodomain-like superfamily protein | GO:0003677 |  |  |  |
| *MDP0000264347* | *AT1G07530.1* | -1.0299 | SCARECROW-like 14 | unknown | GRAS |  |  |
| *MDP0000575908* | *AT5G66770.1* | -1.2127 | GRAS family transcription factor | unknown |  |  |  |
| *MDP0000256486* | *AT5G66770.1* | -1.2644 | GRAS family transcription factor | unknown |  |  |  |
| *MDP0000248279* | *AT4G34610.1* | 1.1121 | BEL1-like homeodomain 6 | GO:0003700,GO:0043565,  GO:0006355,GO:0003677,  GO:0005634 | HB |  |  |
| *MDP0000564897* | *AT2G22430.1* | -1.0891 | homeobox protein 6 | GO:0003700,GO:0043565,  GO:0006355,GO:0003677,  GO:0005634 |  |  |  |
| *MDP0000141097* | *AT4G36870.1* | -1.2456 | BEL1-like homeodomain 2 | GO:0003700,GO:0043565,  GO:0006355,GO:0003677,  GO:0005634 |  |  |  |
| *MDP0000249034* | *AT2G23760.1* | -1.2570 | BEL1-like homeodomain 4 | unknown |  |  |  |
| *MDP0000135540* | *AT5G06710.1* | -1.2814 | homeobox from Arabidopsis thaliana | GO:0003700,GO:0043565,  GO:0006355,GO:0003677,  GO:0005634,GO:0006351,  GO:0016563 | HB |  |  |
| *MDP0000736852* | *AT4G40060.1* | -1.7122 | homeobox protein 16 | GO:0003700,GO:0043565,  GO:0006355,GO:0003677,  GO:0005634 |  |  |  |
| *MDP0000256797* | *AT5G26040.2* | -1.6300 | histone deacetylase 2 | unknown | HDA |  |  |
| *MDP0000319456* | *AT2G26150.1* | 1.4554 | heat shock transcription factor A2 | GO:0003700,GO:0043565,  GO:0006355,GO:0005634 | HSF |  |  |
| *MDP0000212925* | *AT5G48670.1* | 4.0096 | AGAMOUS-like 8unknown | GO:0003677,GO:0046983,  GO:0043565,GO:0006355,  GO:0005634,GO:0003700 | MADS box |  |  |
| *MDP0000167107* | *AT1G25340.1* | -1.2340 | myb domain protein 116 | GO:0003677 | MYB domain |  |  |
| *MDP0000653903* | *AT1G09540.1* | -1.7063 | myb domain protein 61 | GO:0003677 |  |  |  |
| *MDP0000237396* | *AT4G18020.1* | -1.3396 | CheY-like two-component responsive regulator family protein | GO:0000156,GO:0000160,  GO:0006355,GO:0003677 | Psudo ARR |  |  |
| *MDP0000933110* | *AT5G49700.1* | -1.0235 | Predicted AT-hook DNA-binding family protein | unknown | putative transcription regulator |  |  |
| *MDP0000319266* | *AT1G58100.1* | -1.3619 | TCP family transcription factor | unknown | TCP |  |  |
| *MDP0000238683* | *AT5G23280.1* | -1.4438 | TCP family transcription factor | unknown |  |  |  |
| *MDP0000429824* | *AT3G10040.1* | -1.1257 | sequence-specific DNA binding transcription factors | unknown | Trihelix, Triple-Helix |  |  |
| *MDP0000209313* | *AT5G28300.1* | -1.7163 | Duplicated homeodomain-like superfamily protein | unknown |  |  |  |
| *MDP0000202548* | *AT3G26935.1* | 1.6660 | DHHC-type zinc finger family protein | GO:0008270 | unclassified |  |  |
| *MDP0000305455* | *AT1G07360.1* | 1.1391 | CCCH-type zinc fingerfamily protein with RNA-binding domain | GO:0003676,GO:0008270,  GO:0016491,GO:0055114 |  |  |  |
| *MDP0000220601* | *AT3G51950.1* | -1.1413 | Zinc finger (CCCH-type) family protein / RNA recognition motif (RRM)-containing protein | GO:0003676,GO:0008270 |  |  |  |
| *MDP0000243086* | *AT3G51950.1* | -1.5677 | Zinc finger (CCCH-type) family protein / RNA recognition motif (RRM)-containing protein | GO:0003676,GO:0008270 |  |  |  |
| *MDP0000134105* | *AT2G47260.1* | -1.8046 | WRKY DNA-binding protein 23 | GO:0003700,GO:0043565,  GO:0006355 | WRKY domain |  |  |
| *MDP0000127976* | *AT2G47260.1* | -1.9014 | WRKY DNA-binding protein 23 | GO:0003700,GO:0043565,  GO:0006355 |  |  |  |
| *MDP0000652760* | *AT2G47260.1* | -2.3490 | WRKY DNA-binding protein 23 | GO:0003700,GO:0043565,  GO:0006355 |  |  |  |
| *MDP0000708692* | *AT2G38470.1* | -2.4478 | WRKY DNA-binding protein 33 | GO:0003700,GO:0043565,  GO:0006355 |  |  |  |
|  |  |  |  |  |  |  |  |
