# Supplementary material for: Extreme Hypoxic Conditions Induce Selective Molecular Responses and Metabolic Reset in Detached Apple Fruit
Source: Front Plant Sci. 2016 Feb 16;7:146. doi: 10.3389/fpls.2016.00146 (PMC4754620; doi:10.3389/fpls.2016.00146)
Supplement: Supplementary file 8 [file Image1.PDF]

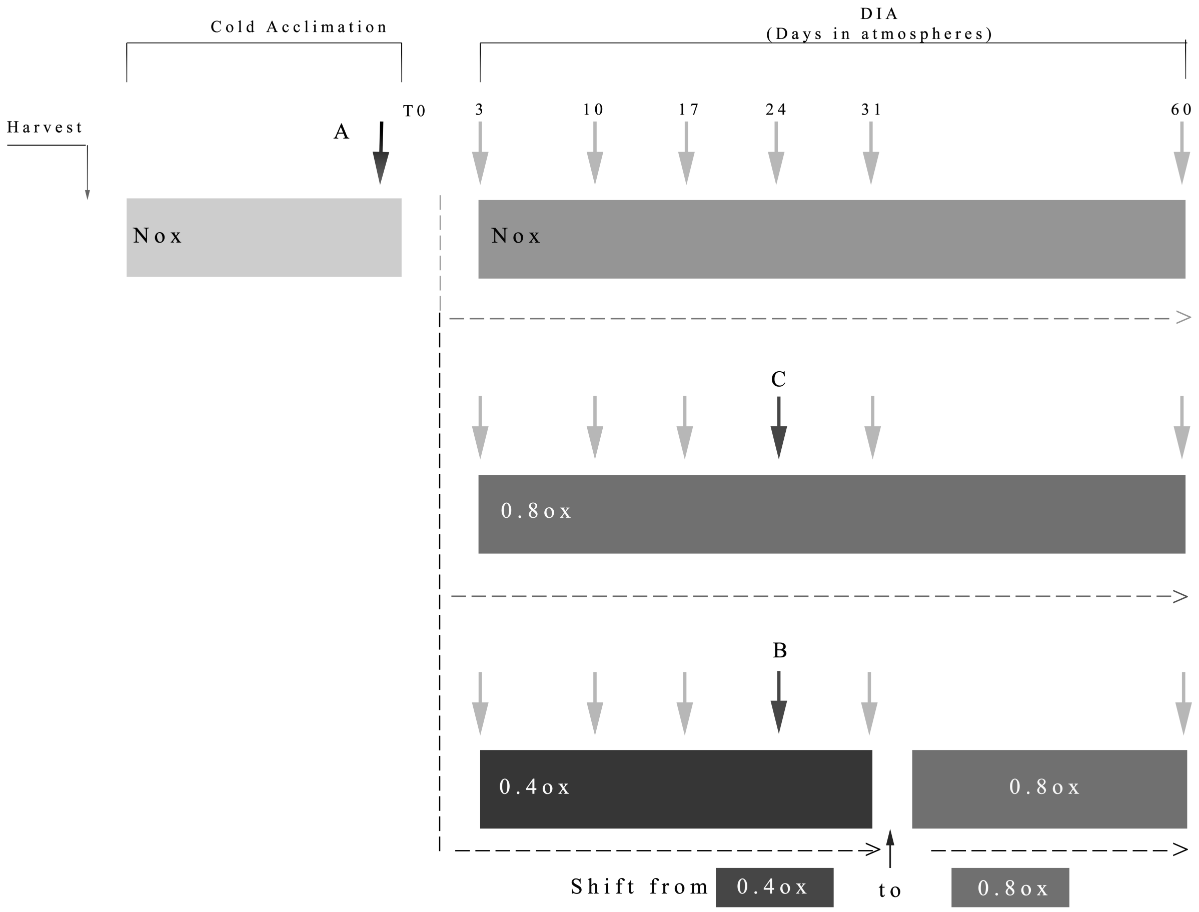

Cukrov et al. supplementary material.

Figure S1 Sample collection of apples subjected to different oxygen regimes. Numbering on the top show the days after the application of the different oxygen regimes (DIA, days in atmospheres) indicated by dotted arrows. T0 represents the start of the experiment with the application of low oxygen levels (0.8 and 0.4 kPa for 0.8ox and 0.4ox samples, respectively) after three days of cold acclimation. The control is represented by apples maintained under normoxic conditions (Nox). Vertical arrows show sampling points for all experimental conditions: samples used for RNAseq analyses are indicated by bold capital letters on the top of black arrows. For the 0.4ox sample, the shift from 0.4 kPa to 0.8 kPa oxygen is indicated by a vertical arrow and was applied at 30 DIA, one day before sampling on day 31.
