# Supplementary figures and images for: Extreme Hypoxic Conditions Induce Selective Molecular Responses and Metabolic Reset in Detached Apple Fruit
Source: Front Plant Sci. 2016 Feb 16;7:146. doi: 10.3389/fpls.2016.00146 (PMC4754620; doi:10.3389/fpls.2016.00146)

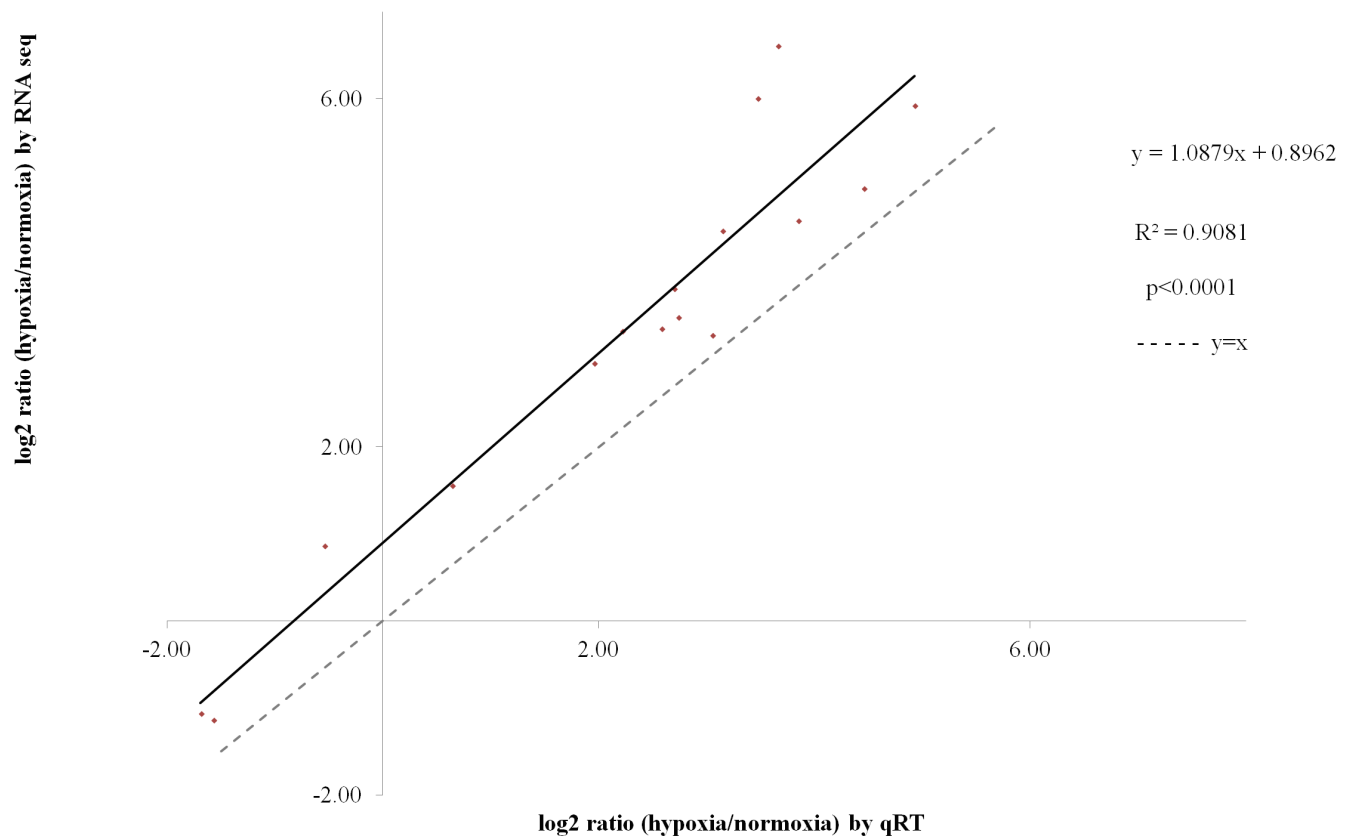

**Cukrov et al. supplementary material.**

**Figure S2** Pearson correlation between RNA-Seq and RT-qPCR

Supplement: Supplementary file 9 [file Image2.PDF]
